# Perioperative Patient Blood Management: Evidence-Based Strategies for Surgeons and Anesthesiologists: A Narrative Review

**DOI:** 10.3390/jcm15083017

**Published:** 2026-04-15

**Authors:** Taxiarchis Konstantinos Nikolouzakis, Epameinondas Evangelos Kantidakis, Richard Crawford, Riaan Pretorius, Orfeas Nikolaos Zaimakis, Emmanuel Chrysos

**Affiliations:** 1 Department of General Surgery, University General Hospital of Heraklion, 71110 Heraklion, Greece; 2 General Practice and Family Medicine, Venizeleion General Hospital, 71409 Heraklion, Greece; 3 Department of Surgery, School of Clinical Medicine, Faculty of Health Sciences, University of the Witwatersrand, Johannesburg 2193, South Africa; richard.crawford@wits.ac.za; 4 Trauma Surgery Unit, Department of General Surgery, Chris Hani Baragwanath Academic Hospital, Johannesburg 1864, South Africa

**Keywords:** patient blood management, perioperative anemia, tranexamic acid, viscoelastic testing, transfusion management

## Abstract

Patient Blood Management (PBM) has evolved from a transfusion-centered practice to a structured, patient-focused perioperative strategy aimed at improving surgical outcomes while preserving blood resources. In the operating room, where bleeding risk is anticipated and modifiable, PBM requires proactive intervention rather than reactive transfusion. This review synthesizes current evidence on perioperative blood conservation strategies specifically relevant to surgeons and anesthesiologists. Preoperative optimization begins with systematic identification and correction of anemia, most commonly iron deficiency, using appropriately timed oral or intravenous iron therapy and, in selected cases, erythropoiesis-stimulating agents. Careful management of anticoagulant and antiplatelet therapies, early recognition of acquired or inherited coagulopathies, and protocol-driven reversal strategies further reduce perioperative hemorrhagic risk. Intraoperatively, blood conservation depends on meticulous surgical technique, respect for anatomical planes, minimally invasive approaches, and the judicious use of advanced energy devices and topical hemostatic agents. Pharmacologic interventions—particularly tranexamic acid administered with appropriate timing and dosing—have demonstrated consistent reductions in blood loss and transfusion requirements across multiple surgical disciplines. Goal-directed coagulation management guided by viscoelastic testing allows targeted correction of specific hemostatic deficits while minimizing unnecessary blood product exposure. Acute normovolemic hemodilution and intraoperative cell salvage provide additional benefit in selected high-blood-loss procedures. Collectively, these multimodal strategies shift perioperative care from product-driven transfusion toward physiology-based blood conservation. When embedded within institutional protocols and supported by multidisciplinary collaboration, perioperative PBM reduces transfusion exposure, decreases morbidity, shortens hospital stay, and promotes sustainable stewardship of blood resources without compromising patient safety.

## 1. Introduction

Blood management in surgical patients has undergone a profound transformation over the past two decades. What was once considered a routine and largely benign supportive therapy, transfusion of allogeneic blood is now recognized as a complex intervention with significant implications for perioperative outcomes [1]. For many years, liberal transfusion practices were broadly accepted despite lacking robust scientific validation. As outcome data accumulated, however, this approach was increasingly called into question. This evolution has given rise to the systematic paradigm of Patient Blood Management (PBM), a multimodal, evidence-based standard of care specifically designed to optimize patient outcomes while promoting judicious and responsible utilization of blood resources [2]. In the meantime, a growing body of literature has provided high-quality description of the potential risks associated with transfusion. These include immunomodulatory effects such as Transfusion-Related Immunomodulation (TRIM) [3]; serious pulmonary complications including Transfusion-Associated Circulatory Overload (TACO) and Transfusion-Related Acute Lung Injury (TRALI) [4]; hemolytic reactions; and the markedly reduced but not absent risk of pathogen transmission. However, apart from the above mentioned clinical risks associated with allogeneic transfusion, the cumulative economic burden it is now a well-recognized issue. Direct product costs, as well as substantial indirect costs associated with extended hospital and ICU stays and the management of transfusion-related complications, further underscore the urgent necessity for comprehensive conservation strategies [5]. At a systems level, the sustainability of the blood supply remains an ongoing concern, particularly in aging populations requiring increasingly complex surgical interventions [6]. These converging clinical and logistical pressures led to a critical reappraisal of transfusion practice. Landmark randomized trials, including the Transfusion Requirements in Critical Care (TRICC) study [7] and Transfusion Requirements in Cardiac Surgery (TRICS) [8] trials, provided robust, high-quality evidence establishing that a restrictive transfusion strategy proves to be equivalent or superior to a liberal approach across most patient populations. Ongoing research continues to refine individualized thresholds across surgical subpopulations [9], yet the shift from empirical triggers to evidence-based restraint remains one of the most important developments in modern perioperative medicine. From this shift emerged the structured concept of Patient Blood Management (PBM), now endorsed by major international health authorities, including the World Health Organization [6]. PBM is built on three interrelated pillars—optimization of red blood cell mass, minimization of blood loss, and enhancement of anemia tolerance—supported by a coordinated bundle of perioperative strategies [10,11]. In clinical practice, these pillars correlate with preoperative anemia treatment, careful management of antithrombotic therapy, intraoperative antifibrinolytics, precise surgical hemostasis, and goal-directed coagulation monitoring [12,13]. Decisions made during this perioperative period have lasting consequences, influencing postoperative recovery, complication rates, and the need for critical care support [14]. This review examines the evidence supporting multimodal perioperative PBM with particular emphasis on its practical implementation in surgical and anesthetic practice. By integrating current data within the three-pillar framework, we aim to provide clinicians with a coherent and clinically grounded approach to blood management that prioritizes patient safety while ensuring responsible stewardship of a vital and limited resource. To support this objective, we conducted a structured literature search in PubMed, Scopus, and the Cochrane Library for articles published between January 1990 and December 2025. Search terms included “patient blood management,” “perioperative anemia,” “tranexamic acid,” “viscoelastic testing,” “transfusion,” “massive transfusion protocol,” “damage control resuscitation,” and related keywords. Priority was given to randomized controlled trials, meta-analyses, systematic reviews, and major clinical practice guidelines. Reference lists of included articles were also hand-searched to identify additional relevant studies. Given the narrative nature of this review, we prioritized high-quality evidence when available and noted areas where evidence remains limited or conflicting. However, this manuscript has inherent methodological limitations. First, our search strategy did not follow a PRISMA protocol, introducing potential selection bias. Second, we did not perform formal quality assessment of included studies or quantitative synthesis of results. However, given the broad, multidisciplinary scope of perioperative patient blood management—spanning preoperative anemia treatment, intraoperative surgical technique, coagulation monitoring, transfusion protocols, and special populations—a narrative synthesis remains an appropriate format to provide clinically actionable guidance for surgeons and anesthesiologists. Readers seeking quantitative effect estimates are referred to the individual meta-analyses and randomized trials cited throughout this review. Table 1 summarizes the key interventions from preoperative assessment through to postoperative recovery following the three pillar approach.

## 2. Preoperative Optimization: The Foundation of Surgical PBM

### 2.1. Anemia Management

Anemia is defined as hemoglobin < 13 g/dL in men and <12 g/dL in non-pregnant women, and its preoperative correction constitutes the first crucial step in PBM. Increased risk of infection, length of stay, perioperative mortality and overall morbidity are strongly associated with untreated anemia, which is present in up to one-third of adult surgical patients.

#### 2.1.1. Iron Deficiency Anemia

The most common cause of preoperative anemia is iron deficiency anemia (IDA), which is seen in 40–60% of cases, with slight variations among different surgical populations and related comorbidities [15,16]. In everyday clinical practice, IDA manifests in two different ways. First, absolute iron deficiency reflects exhaustion of iron stores, with reduced serum ferritin. Second, functional iron deficiency occurs in inflammatory settings, where hepcidin overexpression traps iron in storage sites, making it unavailable for erythropoiesis [40]. Both mechanisms matter clinically. Insufficient iron weakens bone marrow response and reduces the patient’s ability to tolerate surgical blood loss. Anemia severity, timing, inflammation and factors related to the patient, should dictate the choice between oral or intravenous (IV) iron therapy [41]. Oral iron has long been considered first-line treatment because of its low cost and ease of administration [42]. For elective surgery with moderate-to-high expected blood loss, we advise initiating oral iron supplementation at least 6–8 weeks preoperatively. This measure can increase hemoglobin levels by approximately 1–2 g/dL [43]. However, its practical constraints are notable. Up to 40% of patients show poor compliance due to gastrointestinal side effects such as nausea, constipation, abdominal discomfort, and diarrhea [44]. Furthermore, intestinal iron absorption is compromised in patients with chronic inflammation, malignancy, obesity, or other inflammatory conditions. In these settings, oral supplementation is often unsuccessful [45]. Fractional absorption can be improved via intermittent dosing schedules, which also partially alleviate gastrointestinal symptoms, offering a more fitting strategy for some patients [46]; however, this approach does not solve the initial problem created by inflammation. That being said IV iron has become a practical solution among surgical patients with moderate-to-severe anemia or with a short preoperative time interval. It is particularly befitting in instances of impaired iron mobilization, poor oral tolerance, or for procedures with a narrow timeframe for optimization (usually 4–6 weeks) [19,47]. Formulations such as ferric carboxymaltose and ferric derisomaltose allow infusion of 1000–1500 mg in 15–20 min. Severe hypersensitivity reactions are rare when protocols are followed [48,49]. IV iron bypasses the intestinal barrier and delivers iron directly to the marrow. This ensures complete bioavailability and provides a reliable, rapid hemoglobin response [50]. This is consistent with the findings of a 2025 meta-analysis which corroborated that IV iron prior to surgery surpasses oral iron in increasing hemoglobin levels, especially in individuals with chronic kidney disease, cancer, or inflammatory bowel disease [51]. Likewise, a 2026 cost-effectiveness analysis in women with increased hemorrhage due to menstruation—a causal factor of IDA—reported that initial therapy with single-dose IV iron was proven to be beneficial by improving life quality and limiting follow-up healthcare services [52]. Randomized trials and pooled analyses have also found a decrease in perioperative erythrocyte transfusion and indications of reduced postoperative infection risk and shorter hospitalization in specific patient groups [53,54]. Nevertheless, the relationship between hemoglobin correction and meaningful clinical outcomes is not always straightforward. The PREVENTT trial (487 patients undergoing major abdominal surgery) found that IV iron given 10–42 days preoperatively improved hemoglobin but did not reduce transfusion rates or improve outcomes [20]. Timing, surgical setting, and patient selection determine whether IV iron provides clinical benefit. Concerns about safety also necessitate further debate. Serious hypersensitivity reactions occur rarely with modern IV iron compounds if proper protocols are adhered to [49,55]. However, concerns regarding infection remain relevant. A 2021 systematic review and meta-analysis reported a modest but statistically significant increase in infection risk with IV iron compared to oral or no iron supplementation [56]. Although the individual randomized controlled trials lacked a uniform definition for infection, these findings highlight the need for vigilance, especially in patients with ongoing infection or those undergoing interventions with increased microbial burden. Recent international PBM guidelines therefore recommend a tailored approach, favoring IV iron as first-line therapy in fast-track surgical pathways and in patients unlikely to respond to oral supplementation [57].

#### 2.1.2. Erythropoiesis-Stimulating Agents (ESAs): Evidence, Risks, and Perioperative Protocols

Erythropoiesis-stimulating agents (ESAs), such as epoetin alfa and darbepoetin alfa, are recombinant analogues of endogenous erythropoietin that stimulate red blood cell production. In the context of the first pillar of PBM (erythrocyte mass optimization) ESAs offer a potential modality of increasing hemoglobin in specific populations. Their greatest value is seen among patients in whom oral iron intake is not sufficient for anemia correction, especially prior to surgery where exposure to transfusion is undesirable [21]. Nevertheless, they have specific indications and certain risks must also be taken into account, prior to their application, establishing them as supportive therapies within an elaborate, evidence-based strategy [12,21]. Evidence from randomized trials and meta-analyses indicates that perioperative ESA use, especially when combined with iron supplementation, can reduce transfusion requirements across a range of surgical specialties, including major orthopedic, cardiac, and oncologic procedures [58]. For example, in orthopedic surgery, where pre-existing anemia is frequent and hemorrhage is usually expected, preoperative use of ESAs has been correlated with reduced rates of allogeneic transfusion [58]. The most consistent results occurred with higher-dose protocols (e.g., 600 IU/kg, once a week) started three to four weeks preoperatively along with concomitant IV iron to avoid iron sequestration and promote efficient red blood cell production. Despite these advantages, ESAs are not broadly used. Their usage is limited due to safety issues and lack of robust data in reducing mortality or morbidity. The primary concerns are caused by a dose-related increase in cardiovascular and thromboembolic events, such as venous thromboembolism, myocardial infarction and stroke [59]. These risks increase with higher hemoglobin targets (especially above 13 g/dL) and in patients with cardiovascular disease. In the oncologic setting, further caution is required. ESA use in patients undergoing curative cancer treatment has been associated with decreased survival and possible tumor progression, leading to general contraindication in this context. Accordingly, contemporary guidelines, including those from the European Society of Anaesthesiology and Intensive Care (ESAIC) and the European Society of Cardiology (ESC), recommend a restrictive and individualized approach to ESA therapy [12,21]. ESAs are not advised for routine perioperative use. They may be considered in patients with substantial anemia (for example, hemoglobin < 10 g/dL), in individuals with limited transfusion options such as Jehovah’s Witnesses, or in those facing procedures with very high anticipated blood loss where the benefits of transfusion avoidance are judged to outweigh thrombotic risk [60]. When ESAs are used, strict adherence to best-practice protocols is essential. Iron supplementation—preferably intravenous—must accompany therapy to ensure substrate availability for erythropoiesis. Hemoglobin targets should remain conservative, generally within the 10–12 g/dL range, avoiding supraphysiologic correction. In addition, appropriate pharmacologic and mechanical thromboprophylaxis is mandatory to mitigate prothrombotic effects.

#### 2.1.3. Nutritional Optimization: Vitamin B12, Folate, and Other Hematinics

**Figure 1:**
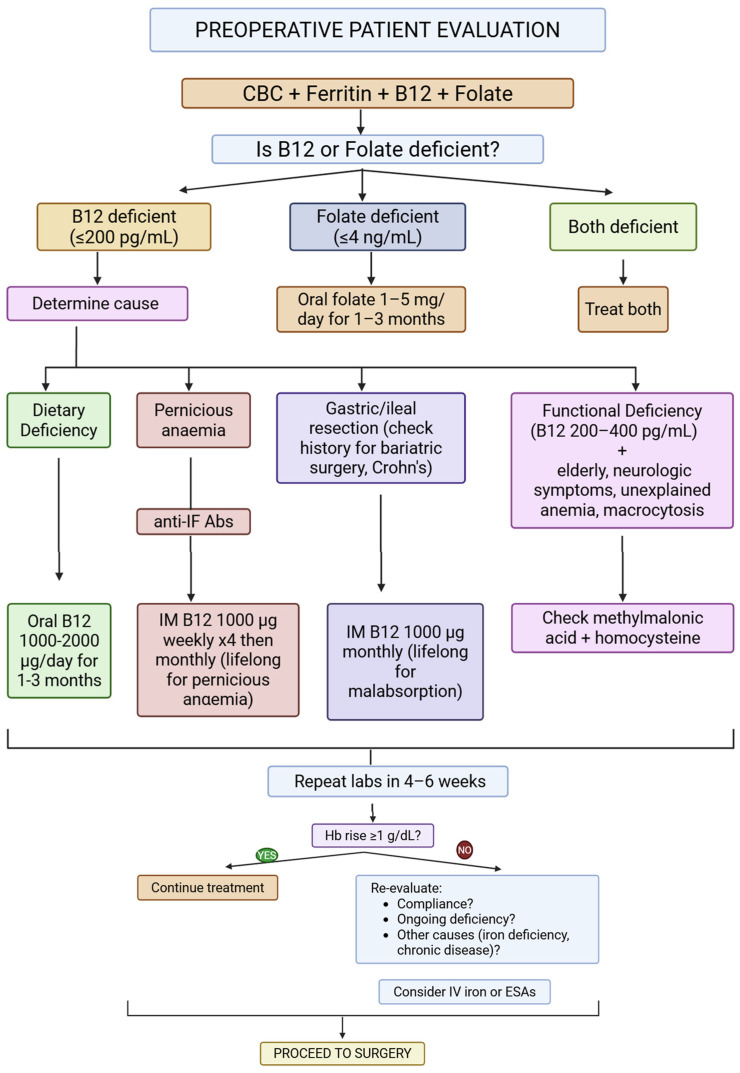
Clinical Algorithm for Preoperative Vitamin B12 and Folate Deficiency Management (CBC, complete blood count; ESA, erythropoiesis-stimulating agent; Hb, hemoglobin; IF, intrinsic factor; IM, intramuscular; IV, intravenous).

Iron is central to erythropoiesis, but it does not act in isolation. Effective red blood cell production depends on adequate availability of vitamin B12 (cobalamin) and folate (vitamin B9), both of which are essential for DNA synthesis during erythroid maturation. Deficiency in either nutrient results in ineffective erythropoiesis, macrocytosis, and shortened red cell survival. These deficiencies frequently coexist with iron deficiency, particularly in elderly patients, those with malnutrition, and individuals with chronic gastrointestinal disease affecting absorption [18,61]. For this reason, evaluation of preoperative anemia should extend beyond hemoglobin and ferritin measurements to include serum B12 and folate levels. Failure to identify these deficiencies may lead to incomplete hematologic response despite appropriate iron therapy, prolonging anemia and increasing transfusion risk [62]. Folate deficiency is usually straightforward to correct with oral supplementation. Vitamin B12 deficiency requires attention to the cause. Oral therapy suffices for dietary deficiency, but parenteral administration is needed for malabsorptive states (pernicious anemia, gastric/ileal resection, Crohn’s disease) [17]. Interpretation of laboratory values can be challenging in older adults. Functional deficiency may be present despite serum B12 levels within the lower end of the reference range. Elevated methylmalonic acid and homocysteine concentrations, particularly in the appropriate clinical context, may signal clinically relevant deficiency even when serum levels appear “normal” [62]. Careful assessment is therefore required in geriatric surgical populations. Certain patient groups warrant particular vigilance. Individuals who have undergone bariatric procedures, especially Roux-en-Y gastric bypass, are at high risk of multiple micronutrient deficiencies due to altered gastrointestinal anatomy and impaired absorption [18]. Persistent iron and B12 deficiencies are common despite routine multivitamin supplementation, necessitating targeted and often lifelong replacement strategies [17,18,61]. Beyond specific hematinic deficits, global malnutrition also carries significant implications for surgical outcomes. Protein-calorie malnutrition has been independently associated with higher rates of postoperative complications, impaired wound healing, and reoperation, particularly in elderly orthopedic populations [22]. Although isolated B12 or folate deficiency may not always correlate directly with early postoperative adverse events, it frequently exists within the broader context of nutritional decline that compromises recovery [22]. Emerging data also suggest that untreated B12 or folate deficiency may influence postoperative neurocognitive recovery. Elevated homocysteine levels have been associated with delayed neurocognitive recovery in elderly patients after non-cardiac surgery, highlighting the broader systemic impact of nutritional status [63]. Comprehensive PBM therefore requires multidisciplinary collaboration, including dietetic assessment when appropriate. Correction of iron deficiency alone is insufficient if concurrent micronutrient deficits persist. Systematic screening for and treatment of B12, folate, and global malnutrition strengthens physiologic resilience, supports effective erythropoiesis, and contributes meaningfully to transfusion avoidance and improved surgical outcomes. Figure 1 provides a clinical algorithm for the identification and management of vitamin B12 and folate deficiencies in preoperative patients. Other hematinic deficiencies—including iron deficiency (see Section 2.1.1) and protein-calorie malnutrition (see Section 4.2.3)—are addressed separately, as they require distinct diagnostic and therapeutic approaches.

#### 2.1.4. Special Populations: Chronic Kidney Disease, Cancer, and Inflammatory Bowel Disease

Anemia in patients with chronic kidney disease (CKD), malignancy, or inflammatory bowel disease (IBD) presents distinct pathophysiologic and therapeutic challenges. These populations experience higher rates of transfusion and adverse postoperative outcomes, necessitating tailored PBM strategies [16,64]. In CKD, anemia arises from relative erythropoietin deficiency, chronic inflammation, iron sequestration, and reduced red cell survival [64]. Intravenous iron is therefore the preferred modality for repletion [65]. ESAs may be required but should be used cautiously; higher hemoglobin targets have been associated with increased risks of hypertension, thromboembolism, stroke and cardiovascular events [66]. Current guidance favors conservative targets, generally not exceeding 11–11.5 g/dL, and emphasizes concurrent IV iron to optimize response and reduce ESA dose requirements [65]. Cancer-associated anemia is multifactorial, reflecting iron restriction, marrow suppression, nutritional deficiency, and treatment effects. IV iron has demonstrated superior efficacy compared with oral iron in improving hemoglobin levels and reducing transfusion requirements, including in patients undergoing major oncologic surgery [67,68]. Importantly, available evidence does not demonstrate an increased risk of tumor progression or cancer recurrence associated with perioperative IV iron administration [68]. In contrast, the use of ESAs in cancer patients remains controversial. Meta-analyses and regulatory reviews have linked ESA therapy to increased thromboembolic risk and, in some settings, reduced overall survival, leading to restrictive recommendations for their use [69]. Consequently, ESAs are generally avoided in curative oncologic surgery and are typically reserved for selected patients receiving palliative chemotherapy, where symptom control is the primary goal [69]. Within PBM programs, IV iron represents the principal strategy for anemia correction in cancer patients, while transfusion is reserved for severe or symptomatic anemia [16]. In IBD, anemia is highly prevalent affecting up to one-third of individuals and often recurrent, driven by chronic blood loss, inflammation, malabsorption, and impaired erythropoiesis [70]. Active disease significantly limits oral iron absorption and may exacerbate gastrointestinal symptoms. The pathogenesis is dominated by chronic gastrointestinal blood loss, malabsorption, inflammation-mediated iron sequestration, and reduced erythropoietic response [64]. For this reason, international guidelines recommend IV iron as first-line therapy in most patients with IBD-associated anemia, particularly when disease activity is present, anemia is moderate to severe or there is documented prior intolerance to oral formulations [70,71]. Early correction before surgery is essential to reduce transfusion exposure and support recovery. Across these conditions, the key principle is early recognition and targeted treatment of anemia while minimizing unnecessary transfusion [16,71]. Disease-specific strategies—favoring IV iron where appropriate, applying ESAs selectively and conservatively, and adhering to restrictive transfusion thresholds—are central to improving perioperative outcomes in high-risk surgical patients [16].

### 2.2. Coagulation Optimization

Optimizing coagulation before and during surgery requires more than simply correcting abnormal laboratory values. It demands a structured approach that balances the risks of bleeding and thrombosis while integrating pharmacologic, procedural, and institutional strategies into a structured plan [12,72,73]. Many centers have introduced formal algorithms and restricted formularies for products such as recombinant factor VIIa, factors VIII and IX, and von Willebrand factor to ensure appropriate and cost-effective use [12]. Coagulopathy—whether related to medications, systemic disease, trauma, dilution, or inherited disorders—is a recognized independent predictor of adverse surgical outcomes [74]. The emphasis has therefore shifted from reactive transfusion during hemorrhage to proactive identification and correction of hemostatic abnormalities before bleeding occurs [12,72,75]. This requires structured preoperative assessment, individualized antithrombotic management, and early recognition of occult bleeding disorders.

#### 2.2.1. Anticoagulant/Antiplatelet Management Bridging Protocols

As the mean age of the surgical patients increases, the number of those receiving long-term anticoagulant or antiplatelet therapy for atrial fibrillation, venous thromboembolism, mechanical heart valves, or coronary artery disease increases as well [76]. Inadequate perioperative management of these agents remains a major contributor to bleeding and transfusion [23,77]. A structured assessment of both thrombotic and procedural bleeding risk is therefore essential [78]. Vitamin K antagonists such as warfarin are typically discontinued four to five days before surgery to allow normalization of the international normalized ratio (INR) [76]. The role of “bridging” with low-molecular-weight heparin (LMWH) has been carefully re-evaluated. Evidence from randomized trials, including the BRIDGE study, demonstrated that routine bridging in patients with atrial fibrillation did not reduce thromboembolic events but significantly increased major bleeding [24]. As a result, bridging is now generally reserved for patients at very high thrombotic risk, such as those with mechanical mitral valves or recent thromboembolism [77]. Direct oral anticoagulants (DOACs) (apixaban, rivaroxaban, dabigatran, and edoxaban) have simplified perioperative management because of their predictable pharmacokinetics and shorter half-lives [79]. In most cases, temporary interruption 24–72 h before surgery—adjusted for renal function and procedural bleeding risk—is sufficient, and bridging is not required [79]. The timing of resumption is equally important; premature reinitiation increases bleeding risk, whereas excessive delay raises thrombotic risk [76]. Antiplatelet therapy presents distinct challenges, particularly in patients with coronary stents. Aspirin is often continued in individuals at high cardiovascular risk unless surgical bleeding risk is prohibitive [80]. P2Y12 inhibitors (e.g., clopidogrel, ticagrelor, prasugrel) are usually stopped five to seven days before elective surgery [76]. In patients at high bleeding risk, such as those with drug-eluting stents, shortening dual antiplatelet therapy (DAPT) to just 1 month followed by antiplatelet monotherapy has been shown to reduce bleeding without increasing ischemic risk [76,80]. Table 2 summarizes the most commonly used anticoagulant and antiplatelet agents, their associated hemorrhage risk, discontinuation timing, bridging protocols, and reversal strategies to assist clinicians in making individualized perioperative decisions.

#### 2.2.2. Identification and Reversal of Coagulopathies

Acquired coagulopathies may arise from liver dysfunction, renal failure, sepsis, trauma, massive transfusion, anticoagulant therapy, or dilutional effects. Traditional laboratory tests remain important screening tools. These include prothrombin time, aPTT, platelet count, and fibrinogen concentration. However, they provide only limited insight into global clot dynamics [83]. Viscoelastic testing (TEG/ROTEM) offers real-time assessment of clot dynamics and reduces transfusion requirements compared with conventional laboratory tests [84]. Correction should begin with physiological optimization that is reversal of hypothermia (<35 °C), acidosis (pH < 7.2), hypocalcemia (<1.0 mmol/L), and severe anemia [84]. Thereafter, therapy should be etiology-specific and goal-directed, using coagulation factor concentrates (CFCs) for a targeted approach. Warfarin reversal in urgent situations requires intravenous vitamin K (10 mg) and four-factor prothrombin complex concentrate (PCC). PCC provides a standardized, high concentration of vitamin K-dependent factors in a small infusion volume (typically 20–50 mL) and therefore normalizes INR more rapidly and requires less volume than fresh frozen plasma (FFP), reducing the risk of volume overload and transfusion-related circulatory overload (TACO) [85]. For DOACs, specific reversal agents (idarucizumab for dabigatran and andexanet alfa for factor Xa inhibitors) are preferred when available; four-factor PCC is an accepted alternative [81]. Fibrinogen depletion is common in major hemorrhage and correlates with worse outcomes. The optimal strategy for fibrinogen replacement, however, remains an area of active investigation and clinical debate. Replacement may be achieved using fibrinogen concentrate or cryoprecipitate. Evidence comparing the two remains mixed. A 2025 meta-analysis evaluated early fibrinogen replacement. It found no mortality benefit, no transfusion superiority, and no reduced risk of deep vein thrombosis. The analysis also found no clear advantage of fibrinogen concentrate over cryoprecipitate [86,87]. Current European Society of Anaesthesiology and Intensive Care (ESAIC) guidance on management of severe peri-operative bleeding reflects this uncertainty and recommends that product selection be guided by clinical context and availability [12]. The efficacy of using FFP to correct significant fibrinogen deficits is limited, as the required volumes become impractically large. Tranexamic acid is recommended for suspected hyperfibrinolysis [84]. In patients receiving antiplatelet therapy (aspirin, clopidogrel, ticagrelor, prasugrel), management of bleeding is complex. Platelet transfusion remains the principal intervention, though its effectiveness depends on the agent involved and timing of the last dose [81]. Ticagrelor poses particular difficulty, as circulating drug may inhibit transfused platelets for up to 24 h, often rendering standard transfusion ineffective [82,88]. Point-of-care platelet function testing (e.g., Multiplate, VerifyNow, TEG Platelet Mapping) can assist in determining the degree of inhibition and guiding transfusion decisions. Adjunctive therapies such as desmopressin and tranexamic acid may be used selectively, though high-quality outcome data remain limited. A systematic review found that desmopressin can improve platelet function and bleeding time in patients on antiplatelet therapy, though more clinical trials are needed [82]. Tranexamic acid is also recommended early in the management of severe bleeding in these patients, regardless of antiplatelet status [81,83,89].

#### 2.2.3. Genetic Testing for Bleeding Disorders (e.g., vWD, Hemophilia Carriers)

Inherited bleeding disorders are often underrecognized in surgical populations, particularly in individuals with mild phenotypes or female carriers [90]. Common symptoms prompting evaluation include menorrhagia, easy bruising, recurrent epistaxis, and prolonged bleeding after dental work or surgery [90]. Routine coagulation tests (prothrombin time, activated partial thromboplastin time, platelet count) serve as initial screening tools [91]. Unidentified disorders significantly increase perioperative bleeding risk and lifetime transfusion exposure (a ninefold increased risk of requiring a blood transfusion over a lifetime is expected in this group) [90,92]. This under-recognition is especially pronounced in females, where bleeding symptoms like menorrhagia (heavy menstrual bleeding) may be normalized, yet up to one-third of women with this symptom may have an undiagnosed bleeding disorder [90]. A structured bleeding history, including personal and family history, a physical examination and a review of medications that can affect hemostasis is therefore indispensable [93,94]. Routine coagulation tests may be normal in conditions such as mild von Willebrand disease (VWD) or factor XIII deficiency, necessitating targeted testing when clinical suspicion is high [93]. VWD requires assessment of von Willebrand factor antigen, activity (e.g., ristocetin cofactor), and factor VIII levels, with management tailored to subtype and severity. Desmopressin may suffice in responsive type 1 disease stimulating endogenous VWF release, while VWF-containing concentrates (plasma-derived or recombinant) are required for major procedures to achieve and maintain hemostatic levels for 3 to 14 days, depending on the procedure [90]. Stratifying surgical risk involves assessing the procedure type, baseline VWF/FVIII levels, and the patient’s bleeding history [93]. Antifibrinolytic agents (e.g., tranexamic acid) are useful adjuncts, particularly for mucosal procedures. Female carriers of hemophilia A or B represent a frequently overlooked group. Although often asymptomatic, factor levels may be reduced sufficiently to increase surgical bleeding risk [95,96]. Diagnosis requires both factor level measurement and genetic analysis [97]. Modern next-generation sequencing panels allows for comprehensive evaluation of bleeding-associated genes and facilitate accurate diagnosis, family screening, and reproductive counseling [94,98]. Early recognition of inherited disorders enables appropriate prophylaxis and reduces avoidable hemorrhage, aligning directly with PBM principles.

### 2.3. Autologous Blood Predonation

Within contemporary PBM programs, the role of autologous blood conservation techniques has been redefined and narrowed to selected indications. They include, preoperative autologous blood donation and normovolemic hemodilution, particularly acute normovolemic hemodilution [16].

#### 2.3.1. Preoperative Autologous Blood Donation: Limitations and Modern Indications

Preoperative autologous donation (PAD) involves collecting and storing a patient’s blood before elective surgery. Once widely adopted during periods of concern about transfusion-transmitted infection, PAD is now rarely indicated [16,99]. The practice may induce or worsen preoperative anemia, potentially increasing the likelihood of transfusion (autologous or allogeneic) [99,100]. High wastage rates (ranging between 30% and 50%), and logistical inefficiencies further limit its appeal [100,101]. Current guidance restricts PAD to highly selected circumstances, such as patients with rare blood phenotypes or complex alloimmunization, or those declining allogeneic transfusion for personal or religious reasons [102]. For most patients, correcting anemia rather than pre-emptively withdrawing blood better aligns with PBM principles [103].

#### 2.3.2. Normovolemic Hemodilution: Current Evidence and Protocols

Acute normovolemic hemodilution (ANH) involves removal of whole blood immediately after induction of anesthesia, with simultaneous volume replacement to maintain normovolemia [104]. Its greater advantage compared to PAD lies on the fact that it does not induce preoperative anemia and avoids storage-related degradation of blood components, preserving platelet function and labile coagulation factors for reinfusion [105,106]. The withdrawn blood, containing intact platelets and clotting factors, is reinfused later during surgery [107]. By reducing the hematocrit before surgical bleeding occurs, ANH decreases the total red cell mass lost. Current evidence supports its use in selected high-blood-loss procedures, particularly in cardiac, hepatic, and complex orthopedic procedures, especially when combined with other modalities like antifibrinolytic therapy within a multimodal PBM protocol [107,108,109]. A 2025 analysis reported reduced transfusion odds with ANH, with greater benefit at higher withdrawn volumes [109]. Proper patient selection is essential; candidates should have adequate baseline hemoglobin undergoing procedures with anticipated high blood loss [104,107]. The volume removed must be carefully calculated based on estimated blood volume and target hematocrit to maintain safety. Although operationally demanding, ANH remains a valuable adjunct in specialized centers when integrated into a broader multimodal PBM strategy [105]. Reframed within modern practice, autologous techniques are no longer routine measures but targeted interventions. When applied selectively and supported by structured protocols, they contribute to transfusion reduction while preserving patient safety [108]. However, due to its operational complexity ANH is not recommended as a routine practice but may be considered in specialized centers as part of an integrated program for appropriate patients [110,111].

## 3. Intraoperative Blood Conservation

### 3.1. Anesthetic and Pharmacological Approaches

During this phase, clinicians can directly influence the physiological and hemostatic responses that occur as a result of surgical trauma [112]. The objective is not simply to suppress bleeding indiscriminately, but rather to create controlled physiological conditions that limit blood loss while maintaining adequate tissue perfusion and avoiding unnecessary thrombotic risk. Achieving this balance requires careful, individualized decision-making that takes into account patient comorbidities, the nature and complexity of the surgical procedure, and continuous physiological monitoring [113]. In practice, intraoperative PBM combines several complementary elements. These include precise control of hemodynamics, pharmacological strategies aimed at enhancing hemostasis, and the selective use of advanced hemostatic agents when needed. The effectiveness of these measures depends greatly on their timing and appropriate application—correct drug dosing, administration at the optimal stage of the procedure, and careful patient selection. This approach reflects the broader shift in perioperative medicine away from reactive transfusion toward proactive, physiology-guided management. When applied effectively, these strategies not only reduce transfusion requirements but have also been associated with improved postoperative recovery, fewer complications, and shorter hospital stays.

#### 3.1.1. Controlled Hypotension: Indications, Limits, and Monitoring

Controlled hypotension is a well-established anesthetic technique used to reduce intraoperative blood loss by lowering arterial pressure and thereby decreasing capillary bleeding at the surgical field. From a PBM perspective, it represents a physiologic method of blood conservation when applied in a controlled and carefully monitored manner. The technique can improve surgical visibility and reduce transfusion requirements in procedures such as orthopedic, spinal, head and neck, and major oncologic surgery [113,114]. The benefits of controlled hypotension depend heavily on appropriate patient selection and vigilant intraoperative monitoring. Patients with significant cardiovascular, cerebrovascular, or renal disease may not tolerate reduced arterial pressure because of impaired autoregulation of organ perfusion and are therefore generally poor candidates for this approach [34]. Rather than aiming for a fixed blood pressure threshold, current practice favors individualized blood pressure goals, commonly maintaining the mean arterial pressure approximately 20–30% below the patient’s baseline level [115]. Continuous invasive arterial pressure monitoring is essential to ensure safety and accuracy. In many cases, additional hemodynamic monitoring—including cardiac output measurement—may also be used to ensure that systemic perfusion remains adequate [116]. Clinicians must remain alert to signs of organ ischemia, including electrocardiographic changes, declining urine output, or reductions in regional oxygen saturation measured with near-infrared spectroscopy when available. Importantly, controlled hypotension is typically used only during phases of the operation associated with high bleeding risk and blood pressure is gradually restored before wound closure. Non-pharmacologic measures can also contribute to the strategy; for example, patient positioning such as reverse Trendelenburg has been shown to reduce bleeding in certain procedures, such as rhinoplasty [113].

#### 3.1.2. Antifibrinolytics: Tranexamic Acid (TXA) Dosing, Timing, and Route (CRASH-3, WOMAN Trials)

Tranexamic acid (TXA) is a cornerstone pharmacologic intervention in modern blood management. It exerts its effect by competitively inhibiting the lysine-binding sites on plasminogen and plasmin, thereby preventing fibrinolysis and stabilizing newly formed clots [28]. Over the past two decades, TXA has become widely incorporated into surgical and trauma protocols, with evidence supporting its use in orthopedic surgery, cardiac procedures, obstetrics, and trauma resuscitation [117,118]. Numerous studies have demonstrated that TXA significantly reduces perioperative blood loss and the need for allogeneic transfusion, making it one of the most effective and cost-efficient pharmacological tools within PBM programs [119]. However, its clinical effectiveness depends largely on appropriate timing and dosing. Understanding TXA pharmacokinetics ensures adequate levels when fibrinolysis peaks [120].

Timing of Administration (Trauma Evidence): The importance of early TXA administration was clearly demonstrated in large international trauma trials. The CRASH-2 and CRASH-3 trials showed that IV TXA within three hours of injury reduces bleeding-related mortality, including in traumatic brain injury [29,30]. These findings were reinforced by a recent systematic review and meta-analysis conducted for the Eastern Association for the Surgery of Trauma (EAST) Practice Management Guidelines [121]. Analyzing data from 30 studies, the investigators reported a significant reduction in 24 h mortality when TXA was administered both in prehospital settings (log risk ratio −0.29; 95% CI −0.53 to −0.05; *p* = 0.02) and in hospital (−0.38; 95% CI −0.69 to −0.06; *p* = 0.02). Similar benefits were observed at 30 days. Importantly, the incidence of thromboembolic events did not differ between treatment and control groups, supporting the overall safety of early TXA administration. On the basis of this evidence, the EAST guidelines provide a conditional recommendation for TXA use in trauma patients at risk of significant hemorrhage [121].

Obstetric Evidence (Distinguishing the WOMAN Trials): Evidence for TXA in obstetrics comes from two major trials that addressed different clinical scenarios. The original WOMAN trial demonstrated that TXA administered within three hours of postpartum hemorrhage (PPH) reduces death due to bleeding in women with established PPH [122]. These findings led the World Health Organization to recommend TXA as part of standard management for postpartum hemorrhage. The WOMAN-2 trial, in contrast, examined whether prophylactic TXA could prevent PPH in women with moderate or severe anemia (hemoglobin < 100 g/L) [31]. In this large randomized study involving more than 15,000 women, TXA administered shortly after delivery did not significantly reduce the incidence of clinically diagnosed PPH. Nevertheless, no increase in thromboembolic events was observed, confirming the drug’s favorable safety profile [31]. A subsequent individual patient data meta-analysis involving more than 54,000 women from five trials provided the most comprehensive assessment of TXA in obstetric practice [123]. The analysis showed that TXA reduces the risk of life-threatening postpartum bleeding—defined as death or the need for surgical intervention—without increasing thromboembolic complications [123]. Taken together, these studies highlight a critical pathophysiological principle: TXA is most effective when administered early in the course of bleeding, before extensive hyperfibrinolysis has developed. However, the WOMAN-2 trial demonstrates that ‘early administration’ must be tailored to each patient and clinical context. In women with established PPH, treatment within three hours is life-saving. In anemic women, immediate post-delivery prophylaxis does not prevent PPH.

Surgical Dosing and Administration: In elective surgery, TXA is typically administered as a loading dose of approximately 10–20 mg/kg prior to incision or at the start of cardiopulmonary bypass, often followed by additional boluses or continuous infusion to maintain therapeutic plasma levels [124]. While intravenous administration remains the most common approach, topical and intra-articular applications have gained increasing acceptance, particularly in orthopedic and cardiac surgery. These methods allow high concentrations of TXA to be delivered directly to the surgical field while minimizing systemic exposure. A 2024 meta-analysis in cardiac surgery reported that topical mediastinal TXA reduced postoperative chest drainage by approximately 174 mL without increasing thrombotic complications [125]. Similarly, intra-articular TXA has been shown to reduce postoperative blood loss and transfusion requirements in total joint arthroplasty [126]. For certain urological procedures, such as radical cystectomy, systematic reviews continue to confirm the blood-sparing benefits of both IV and topical TXA routes [117]. The choice of route must be individualized, factoring in the type of surgery, the patient’s thrombotic risk, and the desired balance between systemic and local hemostatic effect.

Safety Considerations: Large randomized trials have consistently demonstrated that TXA, when used at recommended doses, does not significantly increase the risk of thromboembolic complications in most surgical and trauma populations [29]. The EAST meta-analysis similarly found no difference in vaso-occlusive events between TXA and control groups [121]. Joint statements from NAEMSP/ACEP/ACSCOT societies have also concluded that prehospital TXA appears safe when administered appropriately with low risk of thromboembolic events or seizure [127]. Nevertheless, higher doses—particularly those historically used in cardiac surgery—have been associated with postoperative seizures, likely related to TXA’s interaction with inhibitory glycine receptors in the central nervous system [128]. As a result, many institutions now favor lower dosing strategies (e.g., a reduced dose of 50 mg/kg total) and increased vigilance in patients with neurological risk factors.

#### 3.1.3. Pro-Coagulant Agents: Recombinant Factor VIIa, Fibrinogen Concentrate, Prothrombin Complex

The management of severe intraoperative coagulopathy has gradually shifted away from empiric plasma transfusion toward a more targeted strategy based on specific coagulation factor replacement. This change reflects broader developments in PBM, where treatment decisions are increasingly guided by the identification of discrete hemostatic deficits rather than by generalized transfusion protocols [129]. By administering concentrated coagulation factors tailored to the underlying abnormality, clinicians can often restore hemostasis more rapidly while avoiding volume overload associated with conventional plasma products. The success of this approach relies heavily on accurate and timely diagnosis of coagulation abnormalities. Intraoperative use of viscoelastic testing—most commonly thromboelastography (TEG) or rotational thromboelastometry (ROTEM)—has become an important component of this strategy. These technologies provide a dynamic, whole-blood assessment of clot formation and stability, enabling clinicians to identify deficiencies in fibrinogen, platelet contribution to clot strength, or impaired thrombin generation in real time [130]. This information allows therapy to be directed at the specific component of the coagulation cascade that is failing. Among the available agents, fibrinogen concentrate is frequently used as an early intervention in major surgical bleeding. Fibrinogen plays a central role in clot formation, serving as the substrate for fibrin generation and contributing significantly to clot firmness. During major hemorrhage its plasma concentration often declines rapidly, sometimes becoming the first coagulation factor to reach critically low levels (<1.5–2.0 g/L). Fibrinogen concentrate offers several practical advantages compared with cryoprecipitate, including standardized dosing, viral inactivation, rapid reconstitution, and smaller infusion volumes [13]. Clinical studies in cardiac, trauma, and major orthopedic surgery suggest that targeted fibrinogen replacement can reduce perioperative bleeding and limit transfusion requirements when administered according to laboratory or viscoelastic thresholds [131]. Prothrombin complex concentrates (PCCs) represent another important group of coagulation factor products. These plasma-derived concentrates contain varying combinations of the vitamin K-dependent factors II, VII, IX, and X. Their primary indication remains the rapid reversal of vitamin K antagonist therapy, particularly in patients requiring urgent surgery or experiencing significant bleeding [132]. More recently, PCCs have also been used in certain cases of acquired coagulopathy not related to anticoagulant therapy, especially when prolonged prothrombin time suggests a deficiency in these factors. However, evidence supporting routine use in non-anticoagulated surgical bleeding remains limited, and the potential—albeit relatively low—risk of thromboembolic complications requires careful consideration when selecting patients for treatment [133]. Recombinant activated factor VII (rFVIIa) occupies a more restricted role. This potent pro-coagulant promotes rapid thrombin generation at sites of tissue factor exposure, producing a localized “thrombin burst” that can help stabilize clot formation. Because of its strong pro-thrombotic potential and association with arterial thrombotic events such as myocardial infarction or stroke, rFVIIa is generally reserved for salvage therapy in life-threatening hemorrhage that has not responded to conventional measures [134]. Before its administration, reversible contributors to coagulopathy—including hypothermia, acidosis, hypocalcemia, and fibrinogen depletion—should be corrected. In most institutions, the decision to administer rFVIIa is made collaboratively within a multidisciplinary team experienced in the management of massive hemorrhage [135].

### 3.2. Surgical Technique and Technology

Advances in surgical technique and technology have transformed the way intraoperative blood loss is addressed. Rather than focusing solely on treating bleeding once it occurs, modern surgical practice increasingly aims to minimize hemorrhage at its anatomical source [136]. Contemporary surgical blood conservation relies on several complementary approaches. Minimally invasive access reduces tissue disruption and vascular injury, advanced energy devices allow precise sealing of vessels during dissection, and topical hemostatic agents provide localized control of residual bleeding from raw surfaces. In certain clinical settings—such as trauma surgery—specialized techniques have also been developed to manage catastrophic hemorrhage and stabilize patients until definitive control can be achieved. Together, these developments enable surgeons to reduce operative blood loss, limit the inflammatory response associated with extensive tissue trauma, and preserve the patient’s endogenous coagulation mechanisms. The cumulative effect is a decreased reliance on allogeneic blood transfusion and improved perioperative outcomes, including lower complication rates and faster recovery [11]. Beyond general principles, specific surgical maneuvers that reduce blood loss include: (1) early identification and control of feeding vessels before parenchymal transection; (2) use of sequential clamping or stapling for vascular pedicles rather than blind dissection; (3) maintaining a bloodless surgical field through intermittent pedicle clamping (e.g., Pringle maneuver in liver surgery) when appropriate; and (4) routine inspection of the surgical bed after irrigation to identify missed bleeding points before closure. These evidence-based maneuvers, when combined with the technologies discussed below, form a complete intraoperative blood conservation strategy.

#### 3.2.1. Minimally Invasive Surgery and the Significance of Anatomical Plane Dissection

The widespread adoption of minimally invasive surgery (MIS) represents one of the most important surgical contributions to PBM. By replacing large open incisions with small trocar ports, laparoscopic and robotic procedures significantly reduce access-related tissue injury. This reduction in trauma translates directly into lower blood loss, improved visualization, and a decreased likelihood of transfusion [135]. A recent meta-analysis examining outcomes in patients undergoing surgery for locally advanced colon cancer reported that minimally invasive proctectomy was associated with substantially lower estimated blood loss and a markedly reduced risk of intraoperative transfusion compared with open surgery [137]. Another key factor contributing to reduced bleeding in MIS is the careful dissection along embryological anatomical planes since they are typically avascular and allow organs to be separated with minimal disruption of vascular structures. Examples include the mesorectal plane in rectal cancer surgery and retroperitoneal planes used in renal and pancreatic procedures [25,26,138]. Robotic-assisted platforms have further enhanced these capabilities. High-definition three-dimensional visualization and articulated instruments with multiple degrees of freedom enable surgeons to perform delicate dissection with greater precision than is possible with conventional laparoscopy. As a result, several complex oncologic procedures—such as radical prostatectomy or pancreaticoduodenectomy—have shown reductions in estimated blood loss when performed robotically in high volume centers [139,140].

#### 3.2.2. Precision Dissection Tools: Harmonic Scalpel^®^, LigaSure^®^, Thunderbeat^®^

The effectiveness of minimally invasive techniques is closely linked to the development of modern energy-based surgical instruments capable of dividing tissue while simultaneously achieving hemostasis. Devices such as ultrasonic scalpels, bipolar vessel sealing systems, and hybrid instruments have largely replaced traditional clamp-and-tie methods in many procedures. Ultrasonic devices, including the Harmonic Scalpel^®^, use high-frequency mechanical vibration to generate localized heat within tissue. This energy causes protein denaturation and coagulum formation, allowing vessels to be sealed while tissue is divided. A major advantage of ultrasonic technology is the limited lateral thermal spread, typically confined to only a few millimeters. This characteristic is particularly important when operating near delicate structures, such as nerves in thyroid or pelvic surgery [141]. A 2022 systematic review of thyroid surgery concluded that using an ultrasonic device reduced intraoperative blood loss by an average of 25 mL and shortened operative time by 15 min compared to conventional knot tying [27]. Bipolar vessel-sealing systems, such as LigaSure^®^, combine electrical energy with controlled pressure to fuse collagen and elastin within the vessel wall, producing a durable seal capable of withstanding high intraluminal pressures. These devices are especially useful for dividing vascular pedicles or tissue bundles, such as mesenteric vessels during colectomy or the uterine artery during hysterectomy and can appear reliable in sealing vessels up to 7 mm in diameter [142]. More recently, hybrid devices such as Thunderbeat^®^ have integrated both ultrasonic and bipolar technologies into a single instrument. This allows surgeons to alternate between rapid tissue division and reliable vessel sealing without changing tools. Clinical studies suggest that such devices may improve operative efficiency while further reducing blood loss in selected laparoscopic procedures [143].

#### 3.2.3. Topical Hemostats: Fibrin Sealants, Gelatin-Thrombin Matrices, Flowables

Even with meticulous surgical technique, diffuse bleeding from capillary beds or raw tissue surfaces can persist. In these situations, topical hemostatic agents provide an additional layer of control by accelerating clot formation directly at the bleeding site. Fibrin sealants (e.g., Tisseel^®^, Evicel^®^) represent one of the most physiologically based approaches to topical hemostasis. These products mimic the final stages of the coagulation cascade by delivering concentrated fibrinogen and thrombin to the surgical field. When the components mix, a fibrin clot forms almost immediately, creating a mechanical seal that can control slow venous or capillary bleeding. Such sealants are commonly used to reinforce vascular anastomoses or to manage bleeding from parenchymal organs such as the liver [32]. A meta-analysis of liver resection studies demonstrated that fibrin sealant application reduced postoperative drainage volume by approximately 20% and was associated with a lower incidence of clinically significant bilious leakage [144]. Gelatin-thrombin matrices (e.g., Floseal^®^, Surgiflo^®^)provide another widely used option. These flowable materials combine mechanical tamponade with biochemical activation of coagulation. The gelatin granules expand upon contact with blood, exerting pressure within the wound, while the thrombin component rapidly converts endogenous fibrinogen into fibrin [145]. Randomized trials in cardiac surgery have shown that these matrices can reduce postoperative mediastinal drainage by over 100 mL in the first 24 h and lower the need for reoperation due to bleeding [146]. Other materials, including oxidized regenerated cellulose (e.g., Surgicel^®^), act primarily as a scaffold for clot formation while creating a localized environment that promotes hemostasis. These agents are frequently used in neurosurgery and general surgery to control low-grade diffuse bleeding from tissue surfaces.

#### 3.2.4. The Use of Resuscitative Endovascular Balloon Occlusion of the Aorta (REBOA), Permissive Hypotension and Intraoperative Cell Salvage (ICS) in Trauma Surgery

Certain clinical settings require intraoperative strategies that are specifically adapted to the pattern and severity of bleeding encountered in that field. Trauma surgery represents perhaps the clearest example, as uncontrolled hemorrhage remains a leading cause of early mortality. In this context, several adjunctive techniques have been integrated into modern trauma care to support rapid hemorrhage control while remaining consistent with the principles of patient blood management. One such development is resuscitative endovascular balloon occlusion of the aorta (REBOA), which has gained increasing attention in patients with non-compressible torso hemorrhage. This scenario is typically seen in severe pelvic trauma or major intra-abdominal injury, where external compression or rapid surgical exposure may not be immediately feasible. The technique involves the percutaneous insertion of a balloon catheter through the femoral artery, followed by positioning within the descending aorta. Depending on the suspected source of bleeding, the balloon is generally inflated in Zone 1 for abdominal hemorrhage or in Zone 3 for pelvic bleeding. Once deployed, the balloon temporarily occludes distal aortic flow. In doing so, it limits ongoing hemorrhage while simultaneously increasing central arterial pressure, thereby helping to preserve coronary and cerebral perfusion during the initial resuscitation phase. It is important to emphasize that REBOA is not intended as definitive treatment. Rather, it functions as a damage-control adjunct, creating a limited but critical time window during which definitive surgical or endovascular hemostasis can be achieved. When incorporated into structured trauma protocols and used in carefully selected patients, REBOA may reduce blood loss and transfusion requirements while improving short-term survival in individuals who might otherwise deteriorate rapidly from uncontrolled bleeding [147,148,149,150]. Alongside mechanical control of hemorrhage, contemporary trauma resuscitation has also shifted toward a more restrained approach to fluid administration. The concept of permissive hypotension reflects this shift. Instead of rapidly restoring normal blood pressure in the actively bleeding patient, clinicians intentionally accept a lower systolic pressure—commonly around 80–90 mmHg—until definitive hemostasis is achieved. The rationale is largely physiological. Excessive fluid resuscitation can increase intravascular hydrostatic pressure and dislodge newly forming thrombi at sites of vascular injury, thereby worsening hemorrhage. By maintaining a lower but still adequate perfusion pressure, permissive hypotension aims to limit additional blood loss while preserving perfusion of vital organs. Clinical studies, particularly in penetrating trauma, suggest that this more restrictive strategy can reduce mortality, mitigate trauma-induced coagulopathy, and decrease the need for large-volume transfusion [151,152,153]. Intraoperative cell salvage (ICS) represents another important component of blood conservation during major trauma and high-risk surgery. The technique allows recovery of shed blood from the operative field and reinfusion of processed autologous red blood cells. Modern cell-salvage systems continuously aspirate blood, mix it with anticoagulant, and then process it through washing and centrifugation cycles. During this process, saline irrigation removes debris, free hemoglobin, and a substantial proportion of inflammatory mediators before the red blood cells are concentrated for reinfusion. In procedures associated with significant blood loss—such as major orthopedic, vascular, or trauma surgery—ICS can meaningfully reduce exposure to allogeneic blood products. Several studies report reductions in transfusion requirements of approximately 30–50%, although the magnitude of benefit varies depending on the surgical context and the volume of blood loss encountered [33]. The technique is particularly valuable in patients with rare blood types or multiple alloantibodies, where compatible donor blood may be difficult to obtain. Nevertheless, its use remains limited in situations involving gross bacterial contamination or enteric spillage, where reinfusion could introduce infectious risk [33].

### 3.3. Monitoring and Goal-Directed Therapy

Effective PBM during surgery depends not only on the interventions applied but also on the ability to monitor physiological changes in real time and respond accordingly. Modern intraoperative management increasingly relies on goal-directed therapy, in which treatment decisions are guided by dynamic measurements of coagulation status, hemodynamics, and tissue perfusion rather than by empiric transfusion practices [136]. Advances in point-of-care testing and hemodynamic monitoring have played a critical role in enabling this strategy, allowing rapid identification of coagulopathy, hypovolemia, or impaired oxygen delivery [154]. When incorporated into structured treatment algorithms, these tools support more precise interventions, reduce unnecessary exposure to blood products, and improve overall patient outcomes.

#### 3.3.1. Point-of-Care Testing (POCT): TEG/ROTEM vs. Conventional Coagulation Tests

Conventional coagulation tests such as prothrombin time and activated partial thromboplastin time remain widely used but have important limitations in the setting of acute bleeding. These tests analyze plasma rather than whole blood and therefore fail to capture the contributions of platelets, red blood cells, and the dynamic interactions that occur during clot formation in vivo (clot strength, stability or hyperfibrinolysis) [35,155]. Viscoelastic testing (TEG/ROTEM) analyzes whole blood clot formation at the bedside, providing information on clot initiation, strength, and breakdown within 10–20 min [36]. The resulting data allow clinicians to identify the specific component of coagulation that is impaired, whether related to fibrinogen deficiency, platelet dysfunction, or excessive fibrinolysis. The clinical impact of this targeted information can be substantial. For example, reduced clot firmness (e.g., FIBTEM A5 on ROTEM) may indicate the need for fibrinogen supplementation, while abnormalities in clot kinetics may suggest platelet dysfunction or insufficient thrombin generation [36,156]. Meta-analyses show that viscoelastic-guided transfusion strategies reduce red blood cell, plasma, and platelet use. These strategies may also lower complication rates in high-risk surgical populations [157].

#### 3.3.2. Viscoelastic-Guided Transfusion Algorithms

The full value of viscoelastic monitoring becomes evident when the results are integrated into structured treatment algorithms that specify targeted interventions based on defined test abnormalities. These protocols help standardize decision-making during complex bleeding scenarios and can shorten the time required to achieve effective hemostasis [158]. The ITACTIC randomized trial compared viscoelastic-guided resuscitation with conventional test-guided management in bleeding trauma patients [159]. Although the study did not demonstrate a difference in 28-day mortality between the two groups, it provided important insights into clinical practice [160]. Patients managed with viscoelastic algorithms tended to receive interventions more quickly and were more likely to receive targeted treatments based on the underlying coagulation abnormality. Plasma transfusion was also reduced in this group, suggesting that algorithm-based approaches may improve efficiency and limit unnecessary blood product use [161]. Subsequent analyses highlighted an additional challenge: the effectiveness of these strategies depends not only on the availability of advanced monitoring technologies but also on their integration into clinical workflows [162]. In the ITACTIC trial, many patients with identified coagulopathy did not receive complete correction. This finding emphasizes the need for better implementation of and adherence to treatment algorithms [38].

#### 3.3.3. Goal-Directed Fluid Therapy: Restrictive vs. Liberal Approaches

**Figure 2:**
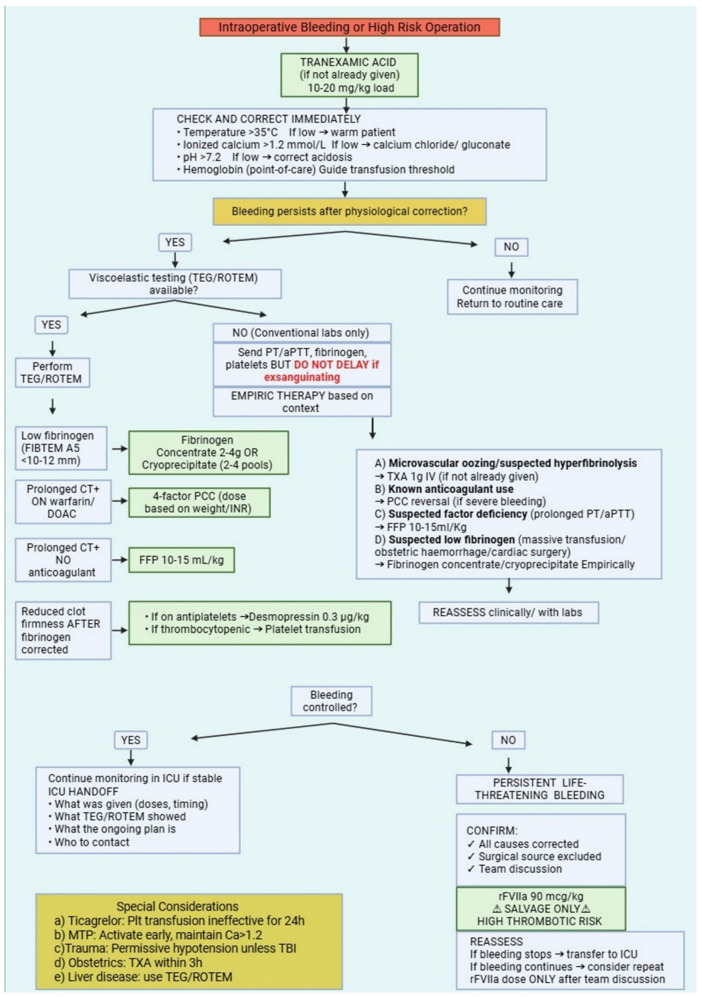
Goal-Directed Intraoperative Bleeding Management Algorithm. Stepwise approach to intraoperative hemorrhage beginning with tranexamic acid administration and physiological optimization (temperature, ionized calcium, pH, hemoglobin). When bleeding persists, viscoelastic testing (TEG/ROTEM) guides targeted therapy: fibrinogen concentrate or cryoprecipitate for low FIBTEM A5 (<10–12 mm); 4-factor prothrombin complex concentrate (PCC) for prolonged clotting time in patients on warfarin or direct oral anticoagulants (DOACs); fresh frozen plasma (FFP) for prolonged clotting time without anticoagulants; and desmopressin or platelet transfusion for platelet dysfunction based on antiplatelet therapy or thrombocytopenia. When viscoelastic testing is unavailable, empiric therapy is guided by clinical context but should not delay intervention in exsanguinating patients. Recombinant activated factor VII (rFVIIa) is reserved for salvage therapy only after all reversible causes are corrected and surgical source control is confirmed, due to high thrombotic risk. Special considerations include ticagrelor (platelet transfusion ineffective for ~24 h), massive transfusion protocols (early activation, 1:1:1 ratios, maintain ionized calcium > 1.2 mmol/L), trauma (permissive hypotension unless traumatic brain injury), obstetrics (tranexamic acid within 3 h of postpartum hemorrhage diagnosis), and liver disease (viscoelastic testing helpful to guide therapy). ICU handoff should document interventions given, test results, and ongoing plan. Multidisciplinary collaboration is essential throughout. Abbreviations: TEG, thromboelastography; ROTEM, rotational thromboelastometry; FIBTEM, fibrinogen thromboelastometry; CT, clotting time; PCC, prothrombin complex concentrate; FFP, fresh frozen plasma; DOAC, direct oral anticoagulant; INR, international normalized ratio; TXA, tranexamic acid; MTP, massive transfusion protocol; TBI, traumatic brain injury; PPH, postpartum hemorrhage; ICU, intensive care unit; rFVIIa, recombinant activated factor VII.

Fluid management represents another key component of intraoperative PBM. Excessive administration of crystalloid solutions can dilute clotting factors and platelets, exacerbate coagulopathy, and increase hydrostatic pressure within the vascular system, potentially worsening bleeding [163]. For this reason, contemporary perioperative care has moved away from liberal fluid strategies toward goal-directed fluid therapy (GDFT). In this approach, fluid administration is tailored to the patient’s physiological response rather than given according to fixed volumes. In short, it is recognized that excessive crystalloid infusion leads to dilution of clotting factors and platelets thus exacerbating the “diamond of death” in trauma (acidosis, hypocalcemia, anemia, and hypothermia). In hand, this accelerates coagulopathy, and increases hydrostatic pressure promoting ongoing hemorrhage [164]. From a PBM perspective, dynamic indicators—such as stroke volume variation, pulse pressure variation, or response to passive leg raising—are preferred for use in assessing fluid responsiveness and guiding resuscitation [165]. By targeting optimal stroke volume while avoiding unnecessary fluid loading, GDFT helps maintain adequate tissue perfusion without excessive hemodilution [166]. Studies in major surgical populations suggest that restrictive or balanced fluid strategies, often combined with vasopressor support when appropriate, may reduce blood loss, decrease transfusion requirements, and improve postoperative recovery [167,168,169]. Importantly, PBM-oriented fluid management does not imply under-resuscitation. Rather, it reflects a commitment to precision: administering the right fluid, in the right amount, at the right time. When integrated with viscoelastic coagulation monitoring and targeted transfusion algorithms, goal-directed fluid therapy completes a comprehensive intraoperative strategy aimed at minimizing blood loss while preserving physiological stability [170]. Table 3 provides a quick-reference guide to intraoperative PBM strategies organized by common clinical scenarios. Figure 2 provides a clinical algorithm integrating these intraoperative monitoring and intervention strategies into a stepwise approach for managing active bleeding or high-risk procedures.

## 4. Special Populations and Scenarios

### 4.1. Massive Transfusion Protocols (MTPs)

Massive hemorrhage remains one of the most time-critical emergencies encountered in acute care medicine. When bleeding becomes severe and uncontrolled, survival often depends on the ability of clinical teams to respond rapidly and in a coordinated manner. Massive transfusion protocols (MTPs) were developed to support this response. Rather than leaving transfusion decisions entirely to ad hoc judgment, these protocols provide a structured pathway that standardizes how blood products are delivered to patients experiencing life-threatening bleeding. In doing so, they reduce variability in practice and ensure that resuscitation strategies remain aligned with the underlying physiology of hemorrhagic shock [171]. The immediate objective is not definitive repair but stabilization—temporizing hemorrhage and addressing trauma-induced coagulopathy so that surgical or interventional control can be achieved [172]. Traditionally, massive transfusion was defined as the administration of at least ten units of packed red blood cells within a 24 h period. While still widely cited, this definition is increasingly viewed as somewhat retrospective. Contemporary practice often adopts more dynamic, rate-based criteria to trigger early activation, such as the need for ≥3 units within one hour, ≥4 units per hour, or replacement of more than 50% of a patient’s estimated blood volume within three hours [172,173]. Determining exactly when to activate an MTP, however, is not always straightforward. Several clinical prediction scores have been proposed, yet even the most widely used tools demonstrate only moderate predictive accuracy. As a result, many trauma systems rely on pragmatic bedside approaches. One such strategy, sometimes referred to as “ABC after 3,” involves administering two or three initial units of red blood cells while closely reassessing the patient’s hemodynamic status and ongoing bleeding. If instability persists, if laboratory or clinical evidence of coagulopathy emerges, or if definitive hemorrhage control is still pending, activation of the full MTP is generally justified [174]. This early escalation reflects growing evidence that survival improves when blood product resuscitation begins promptly—ideally within the first 30 to 36 min following injury.

#### 4.1.1. Balanced Resuscitation Ratios (1:1:1 vs. 1:1:2)

A defining feature of contemporary MTPs is the use of balanced transfusion strategies. Over the past two decades, it has become increasingly clear that hemorrhagic shock is accompanied by rapid and complex disturbances of coagulation. Early trauma-induced coagulopathy can develop within minutes of injury, long before laboratory results become available. Historically, resuscitation often relied heavily on red blood cell transfusion alone. However, this approach failed to address the simultaneous loss of clotting factors and platelets that accompanies severe bleeding [172]. Balanced resuscitation seeks to correct this problem by providing plasma, platelets, and red blood cells in proportions that more closely resemble whole blood. The goal is to restore hemostatic competence early in the resuscitation process rather than reacting only after coagulopathy becomes clinically apparent. Much of the evidence supporting this approach comes from the Pragmatic, Randomized Optimal Platelet and Plasma Ratios (PROPPR) trial, a large multicenter study that compared two commonly used transfusion ratios in patients with severe trauma predicted to require massive transfusion [175,176]. Participants received either a 1:1:1 or 1:1:2 ratio (plasma:platelets:red cells). Overall mortality did not differ, but exsanguination deaths at 24 h were lower in the 1:1:1 group (9.2% vs. 14.6%) [175]. These observations reinforced the importance of providing clotting components early in the resuscitation process [174]. For this reason, many trauma centers now adopt the 1:1:1 strategy as the initial framework for massive transfusion. Nonetheless, the optimal ratio remains an area of ongoing investigation. Some recent analyses suggest that a 2:1 ratio of red blood cells to plasma may achieve similar outcomes in certain settings, leading several consensus groups to emphasize flexibility rather than strict adherence to a single formula. In practice, the guiding principle is to approximate the composition of whole blood during the early phase of resuscitation. Once bleeding begins to stabilize, transfusion strategies should transition toward more individualized management guided by laboratory testing and viscoelastic coagulation monitoring.

#### 4.1.2. Whole Blood Resurgence (Cold-Stored, Low-Titer O Whole Blood)

Perhaps the most notable development in recent years has been the renewed interest in whole blood transfusion, particularly the use of cold-stored, low-titer group O whole blood (LTOWB) [177]. After decades in which component therapy dominated transfusion practice, many trauma systems are revisiting the idea that a single, physiologically balanced blood product may offer important advantages during the earliest stages of resuscitation. LTOWB contains red blood cells, plasma, platelets, and fibrinogen in their natural proportions. As such, it closely mirrors the composition of the blood that is being lost during hemorrhage. Military medical services were among the first to reintroduce this approach on a large scale, reporting encouraging outcomes in combat casualties. More recently, observational studies from civilian trauma centers have suggested that LTOWB may be associated with improved survival and reduced overall use of blood components when compared with conventional component-based therapy [174,177]. Beyond potential clinical benefits, whole blood also offers practical advantages in emergency settings. A single unit automatically delivers a balanced combination of cellular and plasma components, simplifying transfusion logistics and reducing the time required to assemble multiple products. This reduction in “touch time” can be particularly valuable in the chaotic early phase of trauma resuscitation [178]. To allow emergency use across different patient blood groups, most programs rely on low-titer group O whole blood, in which anti-A and anti-B antibody levels are carefully screened. When these titers are sufficiently low, the product can generally be administered safely as an emergency release transfusion. Another advantage is that modern preservation methods allow cold storage while maintaining clinically acceptable hemostatic activity for approximately 21 to 35 days, depending on the anticoagulant solution used. While platelet function does decline during storage, the overall hemostatic efficacy of cold-stored whole blood appears sufficient for early resuscitation [177,178]. In many trauma systems, resuscitation now begins with LTOWB when available, followed by a transition to predefined component packs once the patient’s condition stabilizes and more targeted therapy becomes feasible. To address the remaining uncertainties regarding its effectiveness, several large trials are underway. Among these is the Trauma Resuscitation with Low-Titer Group O Whole Blood or Products (TROOP) trial, a multicenter study designed to compare LTOWB directly with conventional component therapy in severely injured patients [177]. The results are expected to play an important role in shaping future transfusion guidelines.

#### 4.1.3. Damage Control Resuscitation (DCR) Principles

Massive transfusion protocols function most effectively when integrated within the broader framework of Damage Control Resuscitation (DCR). This concept represents a shift away from traditional resuscitation strategies that prioritized rapid normalization of physiology. Instead, DCR focuses on stabilizing the patient through the most critical phase of injury by addressing the immediate threats posed by hemorrhage and coagulopathy [178]. Several principles define this approach. One is the emphasis on hemostatic resuscitation, which discourages the routine use of large volumes of crystalloid fluids. Excessive crystalloid administration can dilute clotting factors, exacerbate acidosis, and contribute to complications such as abdominal compartment syndrome. Consequently, contemporary practice favors early use of blood products while limiting crystalloid exposure [178,179]. Another key concept is permissive hypotension. In patients with truncal hemorrhage but without severe traumatic brain injury (TBI), spinal cord injury, or significant coronary artery disease, a lower systolic blood pressure target (typically 80–90 mmHg, or a mean arterial pressure of 50–60 mmHg) is maintained until surgical control is achieved to avoid dislodging fragile clots [180]. Permissive hypotension should be avoided in patients with TBI (where a higher target, SBP ≥110 mmHg, is recommended to maintain cerebral perfusion), spinal cord injury, pregnancy, and those with significant coronary artery disease [181]. Surgical management also reflects damage-control principles. Damage control surgery (DCS) focuses on rapid control of bleeding and contamination through abbreviated procedures, postponing definitive reconstruction until the patient’s physiology has stabilized. Once hypothermia, acidosis, and coagulopathy have been corrected, a planned return to the operating room allows for completion of the surgical repair [182]. DCR emphasizes prehospital initiation, including immediate hemorrhage control (e.g., tourniquets) and, where available, prehospital transfusion of blood products. A “scoop and run” philosophy minimizes on-scene time, as rapid transport to definitive care is often the highest-value intervention. Adjunctive measures are critical to DCR success. This includes the early administration of TXA, correction of hypothermia (as every 1 °C drop increases transfusion needs by 20%), and proactive replacement of ionized calcium, which should be given during or after the first unit of blood and monitored thereafter to maintain levels above 1.2 mmol/L [183].

#### 4.1.4. Perioperative Transfusion and Postoperative Surgical Complications

The relationship between perioperative allogeneic blood transfusion and postoperative surgical complications has emerged as a critical consideration in surgical quality and patient safety. Numerous large observational studies and meta-analyses have demonstrated that transfusion is independently associated with a higher risk of surgical site infections (SSI), anastomotic leaks, and wound healing disturbances [184,185]. This association is particularly pronounced in colorectal, cardiac, and orthopedic surgery, where transfusion has been linked to a 2- to 5-fold increased risk of infectious complications [184]. The underlying mechanisms are multifactorial but are thought to involve TRIM. Transfusion of allogeneic blood components can suppress innate and adaptive immune responses, impair neutrophil function, and alter cytokine profiles, thereby compromising the host’s ability to mount an effective response against bacterial contamination at the surgical site [3]. This immunological effect may explain the consistent association between transfusion and postoperative infections across diverse surgical populations. Beyond infectious complications, transfusion has been independently associated with an increased risk of reoperation for bleeding or wound complications, prolonged hospital stay, and delayed functional recovery [186]. Importantly, these associations appear to be dose-dependent, with each additional unit of blood products incrementally increasing the risk of adverse outcomes [184]. While causality is difficult to establish definitively given the potential for confounding by indication—sicker patients are more likely to be transfused and also more likely to experience complications—the consistency of findings across diverse surgical populations and the presence of biological plausibility strongly support the view that transfusion itself contributes to postoperative morbidity. These findings provide a compelling rationale for multimodal Patient Blood Management strategies that reduce transfusion exposure. DCR principles—including balanced resuscitation, permissive hypotension, and early use of whole blood or fixed-ratio components—should be viewed not merely as strategies to conserve blood resources, but as direct interventions to improve postoperative surgical outcomes by minimizing the immunological and inflammatory consequences of allogeneic transfusion.

### 4.2. Economic and Quality Outcomes

Beyond its clinical benefits, Patient Blood Management (PBM) also carries significant economic and quality-of-care implications, which are particularly relevant in healthcare systems operating under increasing resource constraints. Blood products are finite and expensive resources, and their costs extend far beyond procurement alone. Expenses accumulate through donor screening, laboratory testing, processing, storage, transport, and administration, as well as through the management of transfusion-related complications when they occur [39]. PBM provides a framework in which cost containment and quality improvement are not competing priorities but rather complementary objectives. By optimizing patients’ hematologic status, minimizing blood loss, and applying evidence-based transfusion thresholds, PBM simultaneously promotes patient safety and more efficient use of healthcare resources. The implementation of multimodal PBM therefore represents not only a clinical advance but also an important economic and quality initiative for healthcare systems [187].

#### 4.2.1. Cost-Effectiveness Analyses of Various Strategies

Economic evaluations of Patient Blood Management consistently show that investments in preoperative optimization and blood conservation strategies can yield substantial financial returns. Importantly, these analyses extend beyond the simple cost of transfused units and instead consider the broader economic consequences of transfusion-related complications, increased resource utilization, and prolonged hospitalization. Multiple studies examining PBM interventions—including preoperative anemia screening and treatment, restrictive transfusion thresholds, and intraoperative blood-sparing techniques—have demonstrated favorable cost-effectiveness profiles across both surgical and critical care populations [188]. At the most basic level, the economic benefit derives from reductions in blood product utilization. This effect has been documented across diverse healthcare systems and surgical specialties. A large systematic review and meta-analysis including 17 studies and more than 235,000 surgical patients found that the implementation of multimodal PBM programs reduced transfusion rates by approximately 39% and decreased the number of red blood cell units transfused per patient by 0.43 units [188]. In clinical environments where transfusion requirements are particularly high, the effect can be even more pronounced. For example, cardiovascular surgery departments that introduced comprehensive PBM programs reported reductions of 21% in red blood cell use and 23.7% in total blood product utilization, translating into substantial institutional cost savings [188]. These reductions become even more meaningful when the full cost of transfusion is considered. Although the direct acquisition cost of leukoreduced red blood cells in the United States averages roughly $208 per unit, the total cost to hospitals—including compatibility testing, processing, storage, and clinical administration—can exceed $1200 per unit according to detailed activity-based costing analyses [39,189,190]. Consequently, even modest reductions in transfusion frequency can translate into considerable financial savings. PBM programs also generate economic value by preventing complications and improving operational efficiency. A cost–benefit analysis based on meta-analytic data estimated that implementing three key PBM interventions—preoperative anemia treatment with iron supplementation, intraoperative cell salvage, and the use of tranexamic acid—cost approximately €129.04 per patient but produced average savings of €150.64 per patient through reductions in transfusion rates, fewer units administered, and shorter hospital stays [188]. The resulting net benefit of €21.60 per patient in that specific analysis demonstrates a potential economic advantage. However, it is crucial to note that cost-effectiveness findings are highly context-dependent and may not generalize across different healthcare systems with varying costs for blood products, labor, and hospital care. When these benefits are extrapolated to larger patient populations, the financial impact becomes even more substantial. Simulation models suggest that comprehensive PBM implementation could generate potential savings of approximately €1,878,000 for every 100,000 patients treated, though these figures are model-dependent and should be interpreted as estimates rather than definite results [189]. The budgetary implications are particularly evident in high-resource surgical specialties. In cardiovascular surgery departments that introduced structured PBM programs, total savings have been reported in the range of 4.2 to 6.6 million Turkish lira (TRY), equivalent to roughly €342,302 following implementation [190]. Importantly, the magnitude of savings depends on how comprehensively PBM principles are applied. Analyses suggest that implementing only the first pillar of PBM—optimization of erythropoiesis and correction of anemia—can generate measurable savings, but the financial benefits increase substantially when all three pillars are implemented simultaneously. In one analysis of cardiovascular surgical practice in Turkey, applying only the first pillar would have prevented approximately 30 complications and saved 4,189,802 TRY, whereas implementing all three pillars was projected to prevent 29 complications while generating savings of 6,174,434 TRY [190]. Although some PBM interventions—such as cell salvage systems or antifibrinolytic therapy—require initial investments in equipment, training, or infrastructure, numerous cost-effectiveness studies demonstrate that these expenditures are offset by reductions in blood product consumption, fewer transfusion-related complications, and lower rates of reoperation due to bleeding [191]. In addition to direct financial savings, PBM may also improve hospital efficiency by reducing the length of hospitalization. Shorter hospital stays free up beds and increase surgical throughput, effectively expanding institutional capacity without requiring additional infrastructure. One analysis estimated that PBM implementation in a cardiovascular surgery department could allow approximately 137 additional procedures to be performed during the same time period, highlighting the potential for significant opportunity-cost savings [188,192].

#### 4.2.2. Impact on Hospital Quality Metrics (LOS, Readmissions, Complications)

Beyond its economic advantages, the adoption of PBM strategies has also been associated with measurable improvements in several key hospital quality indicators. These benefits reflect the fact that reducing exposure to allogeneic blood transfusion can lower the incidence of transfusion-related complications while simultaneously improving perioperative physiological stability. Allogeneic transfusion has been associated with a range of adverse outcomes, including infections, transfusion-related acute lung injury, renal dysfunction, and thromboembolic events [184]. By limiting unnecessary transfusions, PBM strategies reduce the likelihood of these complications, which in turn contributes to shorter hospital stays and improved postoperative recovery. A meta-analysis of 17 studies evaluating comprehensive PBM programs demonstrated a statistically significant reduction in overall hospital length of stay, with a mean decrease of approximately 0.45 days [188]. Although this reduction may appear modest at the individual patient level, its cumulative effect across large patient populations can be substantial. Several mechanisms likely contribute to this improvement. Patients whose anemia is corrected before surgery often exhibit greater physiological resilience and better postoperative recovery. At the same time, blood-sparing surgical techniques and pharmacological interventions reduce intraoperative blood loss and the need for transfusion. Together, these factors decrease postoperative morbidity and accelerate recovery. The impact may be particularly pronounced in cardiac surgery populations, where baseline hospitalization durations are typically longer. Studies evaluating enhanced recovery pathways that incorporate PBM principles have reported reductions in postoperative hospitalization ranging from 1.5 to 2 days [185]. Such improvements not only reduce direct hospitalization costs but also increase bed availability and improve patient flow within the healthcare system. Complication rates also appear to decline following PBM implementation. In the meta-analysis conducted by Althoff and colleagues, PBM programs were associated with a 20% reduction in overall complications (relative risk 0.80; 95% CI 0.66–0.97) and an 11% reduction in mortality (relative risk 0.89; 95% CI 0.81–0.99) [188]. It is important to note, however, that this analysis included predominantly observational and quasi-experimental studies; therefore, causality cannot be firmly established, and the magnitude of benefit may vary across different healthcare settings and patient populations. These improvements were particularly evident for complications linked to transfusion or untreated anemia, including surgical site infections, sepsis, renal failure, myocardial infarction, and stroke [185]. In high-risk specialties such as cardiovascular surgery, even small relative risk reductions can translate into meaningful clinical benefits. One detailed analysis estimated that applying all three PBM pillars in 882 cardiovascular surgical procedures would have prevented approximately 29 major complications [188]. Avoiding these complications not only improves patient outcomes but also reduces the significant costs associated with prolonged intensive care stays, additional procedures, rehabilitation, and subsequent hospitalizations. Evidence regarding readmission rates is somewhat more limited, but available data suggest that PBM may also contribute to reductions in postoperative readmissions. Preoperative optimization—particularly the correction of anemia and nutritional deficiencies—appears to reduce the likelihood of complications that frequently lead to unplanned hospital return [186]. Some observational studies in joint replacement surgery have reported reductions in 30-day readmission rates following the introduction of PBM protocols compared with historical controls [193]. These improvements were particularly evident for readmissions related to surgical site infections, cardiovascular events, and anemia-related complications such as fatigue, falls, or delayed wound healing. Lower preoperative hemoglobin levels are consistently associated with increased perioperative morbidity, although the magnitude of risk varies considerably by patient population and comorbidity burden [194,195].

#### 4.2.3. Elderly: Frailty, Comorbidities, and Individualized Thresholds

Elderly patients represent a rapidly growing proportion of both surgical and critically ill populations. Managing anemia and transfusion decisions in this group presents particular challenges, as advancing age is frequently accompanied by frailty, multiple chronic diseases, and reduced physiological reserve [195,196]. These factors influence both tolerance to anemia and the risks associated with transfusion, making individualized PBM strategies especially important. Frailty reflects diminished physiological reserve, increasing vulnerability to stressors such as surgery, infection, or acute blood loss. Two conceptual models are commonly used to assess frailty. They provide complementary perspectives on vulnerability and risk. Importantly, frailty thresholds may vary across different populations and healthcare settings. For example, a study conducted in Sub-Saharan Africa identified a population-specific frailty threshold of 0.29 using the deficit accumulation model, emphasizing the importance of validating such instruments in different populations and healthcare settings rather than applying universal cutoffs [197]. In elderly surgical patients, frailty is strongly associated with higher rates of postoperative complications, longer hospital stays, and increased mortality [198]. Comorbid conditions further complicate anemia management in older adults. Chronic diseases such as cardiovascular disease, chronic kidney disease, diabetes, and chronic obstructive pulmonary disease are highly prevalent in this population and often contribute to the development of anemia while simultaneously increasing vulnerability to its physiological consequences [199,200]. For example, in patients with coronary artery disease, anemia may precipitate myocardial ischemia at higher hemoglobin levels than would typically cause symptoms in otherwise healthy individuals [201]. At the same time, certain comorbidities can influence the appropriateness of specific PBM interventions [202]. Consequently, effective PBM in elderly patients requires a broader clinical assessment that extends beyond hematologic parameters. Comprehensive geriatric assessment provides a useful framework for this purpose. In addition to evaluating anemia and laboratory values, this approach considers functional capacity, cognitive status, nutritional state, medication burden, and available social support [203]. Such multidimensional evaluation is particularly important given that multimorbidity—defined as the presence of two or more chronic diseases—affects approximately 65% of individuals aged 65 years and older [204]. Individualized transfusion thresholds represent another important component of PBM in older patients. While restrictive transfusion strategies are appropriate for the general population, older patients may benefit from slightly higher thresholds, particularly if symptomatic or with evidence of organ ischemia [205]. Recent evidence has also highlighted the importance of context-specific transfusion strategies in certain high-risk clinical conditions. In patients with acute brain injury, for example, maintaining higher hemoglobin levels may support cerebral oxygen delivery during periods of neurological vulnerability. A 2025 systematic review and meta-analysis by Nguyen and colleagues, which included five randomized controlled trials involving 2399 patients with traumatic brain injury, subarachnoid hemorrhage, or intracerebral hemorrhage, found that a liberal transfusion strategy (hemoglobin threshold ≤ 9–10 g/dL) was associated with a lower risk of unfavorable functional outcomes at six months (relative risk 0.86; 95% CI 0.79–0.94) and a reduced incidence of sepsis compared with restrictive strategies (≤7–8 g/dL) [206]. Similarly, the TRAIN trial demonstrated that transfusing patients with acute brain injury at a hemoglobin threshold below 9 g/dL reduced unfavorable neurological outcomes at 180 days from 73% to 63%, corresponding to an absolute risk reduction of 10% and a number needed to treat of 10 [37]. Fewer cerebral ischemic events were also observed in the liberal transfusion group. These findings suggest that maintaining higher hemoglobin thresholds (e.g., above 9 g/dL) may be considered in selected patients with acute brain injury, although optimal transfusion targets remain uncertain [206,207]. Current consensus from the Neurocritical Care Society and Brain Trauma Foundation continues to emphasize an individualized approach that balances the potential benefits of enhanced oxygen delivery against the risks of transfusion, rather than adopting a universal liberal strategy [208,209]. In practice, transfusion decisions in elderly patients should consider symptoms of anemia, the severity and acuity of blood loss, underlying comorbidities, and the patient’s overall physiological reserve [99]. Randomized trials have addressed transfusion thresholds in elderly patients with cardiac disease. A restrictive strategy (hemoglobin threshold < 8 g/dL) may be associated with increased myocardial injury and cardiovascular events. This was compared to a more liberal approach (threshold < 10 g/dL) [184]. To provide clarity for clinical decision-making, a practical hierarchy can be considered: for most stable, non-cardiac surgical patients, a restrictive threshold of 7 g/dL is appropriate. For patients with known cardiovascular disease or those at high risk for myocardial injury, a threshold of 8 g/dL is often employed based on evidence from cardiac surgery populations. In patients with acute brain injury, a threshold of 8–9 g/dL may be considered based on emerging evidence. Ultimately, in any patient with signs of symptomatic anemia or ongoing end-organ ischemia, the transfusion decision should be individualized and not strictly bound by a numerical trigger. However, these findings must be balanced against the increased risks of transfusion-associated complications in older adults, including TACO, TRALI, and immunomodulatory effects [210]. Multimodal prehabilitation offers particular promise for optimizing elderly patients before surgery. Programs combining physical conditioning, nutritional optimization, anemia correction, and comorbidity management over 2–4 weeks before surgery can improve physiological reserve and potentially mitigate perioperative risk [198]. While evidence specifically in elderly PBM contexts is still evolving, feasibility studies in high-risk, frail elderly patients undergoing major surgery demonstrate that such programs are acceptable and implementable, with good adherence to exercise components though some challenges with nutritional interventions [196]. Integrating PBM into broader prehabilitation frameworks creates a comprehensive optimization strategy that addresses multiple risk factors simultaneously, potentially yielding synergistic benefits for vulnerable elderly surgical patients. Emerging evidence suggests that geriatric-specific PBM protocols that incorporate frailty assessment, individualized transfusion thresholds, and multimodal prehabilitation can reduce transfusion rates by 25–35% in elderly surgical patients while simultaneously decreasing postoperative complications by 20–30% compared to standard care [199]. These protocols emphasize patient-centered outcomes such as functional recovery, cognitive preservation, and quality of life rather than focusing solely on hematological parameters or transfusion rates, aligning with the priorities of older adults undergoing surgery [201].

### 4.3. Limitations and Evidence Gaps in Current PBM Literature

Despite the broad acceptance of PBM principles, several important evidence gaps remain. First, the majority of PBM studies are observational or quasi-experimental in design. High-quality randomized controlled trials directly comparing multimodal PBM programs against conventional care are scarce, particularly for non-cardiac, non-orthopedic surgical populations [188]. Second, the optimal timing and dosing of intravenous iron before surgery remain uncertain. The PREVENTT trial demonstrated that preoperative IV iron improved hemoglobin but did not reduce transfusions or morbidity, highlighting that laboratory correction does not guarantee clinical benefit [20]. Third, while viscoelastic testing reduces transfusion in cardiac and trauma surgery, evidence in other surgical specialties is less robust, and the cost-effectiveness of routine intraoperative TEG/ROTEM in low-risk procedures has not been established [157,159]. Fourth, the role of ESAs in perioperative care remains highly controversial, with thrombotic risks and oncologic safety concerns limiting their use to narrow indications [59,69]. Fifth, frailty-adapted transfusion thresholds have not been tested in prospective randomized trials; current recommendations derive from subgroup analyses and expert consensus [195,210]. Finally, most economic analyses of PBM are context-specific and may not generalize across healthcare systems with different blood product costs, labor expenses, and reimbursement models [189].

### 4.4. Postoperative Blood Conservation Strategies

Postoperative blood loss and transfusion risk do not end in the operating room. Several evidence-based strategies extend PBM into the postoperative period. First, restrictive transfusion thresholds (Hb 7–8 g/dL for stable patients) should be actively maintained, as liberal postoperative transfusion increases complications without improving outcomes [9]. Second, postoperative anemia should be treated with IV iron rather than observation alone; international consensus guidelines recommend early postoperative IV iron to reduce transfusion requirements and support recovery [16]. Third, diagnostic blood draws are a major source of iatrogenic anemia–using pediatric tubes, eliminating “routine daily” labs, and implementing closed blood conservation devices can reduce phlebotomy losses [39]. Fourth, anticoagulation resumption after surgery requires careful timing; premature reinitiation increases bleeding risk, while excessive delay raises thrombotic risk [76]. Finally, early mobilization and mechanical thromboprophylaxis should be employed to reduce venous thromboembolism without increasing bleeding risk. These postoperative measures complete the PBM continuum and should be included in institutional protocols. Table 4 summarizes key clinical takeaways for perioperative blood management.

## 5. Conclusions

Perioperative Patient Blood Management represents a fundamental shift from transfusion-centered practice toward a structured, physiology-driven approach to surgical care. Evidence accumulated over the past two decades demonstrates that systematic identification and treatment of preoperative anemia, careful management of antithrombotic therapy, meticulous surgical technique, and goal-directed intraoperative monitoring can substantially reduce transfusion exposure without compromising patient safety. For surgeons and anesthesiologists, the operating room remains the pivotal environment where these principles are translated into clinical practice. When integrated across the perioperative pathway—from preoperative optimization to intraoperative blood conservation and postoperative transfusion stewardship—PBM supports improved clinical outcomes, including reduced morbidity, shorter hospital stay, and more efficient use of healthcare resources. Ultimately, the successful implementation of PBM depends on multidisciplinary collaboration, institutional protocols, and a shared commitment to evidence-based decision-making. In this context, perioperative blood management should be regarded not as an adjunct to surgical care, but as an integral component of modern perioperative practice.

## Figures and Tables

**Table 1:** The Three Pillars of Perioperative Patient Blood Management (Hb—Hemoglobin, IDA—Iron Deficiency Anemia, IV—Intravenous, ESA—Erythropoiesis-Stimulating Agent, CKD—Chronic Kidney Disease, IBD—Inflammatory Bowel Disease, DOAC—Direct Oral Anticoagulant, LMWH—Low-Molecular-Weight Heparin, INR—International Normalized Ratio, MIS—Minimally Invasive Surgery, ANH—Acute Normovolemic Hemodilution, TEG—Thromboelastography, ROTEM—Rotational Thromboelastometry, PCC—Prothrombin Complex Concentrate, FFP—Fresh Frozen Plasma, TACO—Transfusion-Associated Circulatory Overload, TBI—Traumatic Brain Injury, ICU—Intensive Care Unit, MAP—Mean Arterial Pressure, CV—Cardiovascular, CVA—Cerebrovascular Accident, CABG—Coronary Artery Bypass Grafting, AAA—Abdominal Aortic Aneurysm, DAPT—Dual Antiplatelet Therapy).

Pillar	Phase	Core Objective	Key Clinical Interventions	Clinical Tip
Pillar 1	Preoperative	Optimize red blood cell mass	Screen all surgical patients for anemia (Hb < 13 g/dL men, <12 g/dL women) [15,16]	Don’t forget ferritin, B12, folate–especially in elderly, malnourished, post-bariatric [17,18]
			Ιron deficiency (absolute or functional): oral iron if >6 weeks and no inflammation; IV iron (ferric carboxymaltose, derisomaltose) if <4–6 weeks, functional IDA, oral intolerance [19]	PREVENTT trial reminder: raising Hb doesn’t always improve outcomes–timing and patient selection matter [20]
			ESAs: only in selected cases (Hb < 10, Jehovah’s Witnesses, CKD). Must give with IV iron [21]	Avoid in active cancer. Caution in cardiovascular disease–thrombotic risk is real
			Correct B12/folate deficiencies–especially in elderly, IBD, post-gastric bypass [17,22]	Methylmalonic acid and homocysteine may reveal functional deficiency despite “normal” serum levels
			Manage anticoagulants: stop warfarin 4–5 days preop, DOACs 24–72 h based on renal function/bleeding risk [23]	Bridging with LMWH only for high thrombotic risk (mechanical mitral valves, recent thrombosis)–BRIDGE trial showed harm otherwise [24]
Pillar 2	Intraoperative	Minimize blood loss	Meticulous surgical technique–respect avascular anatomical planes (e.g., mesorectal, retroperitoneal) [25,26]	Minimally invasive approaches reduce blood loss significantly when feasible
			Energy devices: ultrasonic (Harmonic), bipolar vessel sealing (LigaSure), hybrid (Thunderbeat) [27]	Each has different thermal spread profiles–choose based on proximity to heat sensitive structures (e.g., nerves)
			Tranexamic acid 10–20 mg/kg load before incision, consider redosing [28]	CRASH trials: give early (<3 h) for trauma [29,30] WOMAN-2: prophylaxis in anemic women didn’t prevent PPH–context matters [31]
			Topical hemostats: fibrin sealants (Tisseel) for flat surfaces, gelatin-thrombin matrices (Floseal) for irregular/active bleeding [32]	Works synergistically–mechanical tamponade + biochemical activation
			Intraoperative cell salvage–set up for expected blood loss > 500–1000 mL [33]	Avoid if gross contamination. Reduces allogeneic exposure by ~30–50%
			Controlled hypotension (MAP 20–30% below baseline) during high-bleed phases [34]	Only if no CV/cerebrovascular disease. Reverse before closure
			Goal-directed coagulation management–TEG/ROTEM guided therapy [35,36]	Targets specific deficits instead of shooting in the dark with FFP
Pillar 3	Postoperative	Enhance anemia tolerance & avoid unnecessary transfusion	Restrictive transfusion triggers: Hb 7–8 g/dL for most stable patients [9]	Exceptions: acute coronary syndrome, ongoing bleeding, symptomatic anemia
			Higher thresholds in specific populations:	Brain injury: Hb < 9 g/dL (TRAIN trial showed better neurological outcomes at 180 days [37]) Elderly with cardiac disease: consider 8–9 g/dL
			Continue anemia treatment–IV iron, B12, folate if deficient [16]	Transfusion should be coupled with proper treatment of the underlying cause
			Goal-directed fluid therapy–avoid crystalloid overload [38]	Dilutional coagulopathy worsens bleeding
			Minimize diagnostic blood loss–use pediatric tubes, stop “routine daily” labs [39]	Iatrogenic anemia should be expected and prevented

**Table 2:** Perioperative Management of Common Antithrombotic Agents: Hemorrhage Risk and Bridging Protocols [Abbreviations: DOAC, direct oral anticoagulant; INR, international normalized ratio; LMWH, low-molecular-weight heparin; PCC, prothrombin complex concentrate; VTE, venous thromboembolism; DDAVP, desmopressin (1-deamino-8-D-arginine vasopressin), NSAID, non-steroid anti-inflammatory drug].

Drug Class	Haemorrhage Risk	Typical Discontinuation	Bridging Considerations	Reversal
Vitamin K Antagonists (Warfarin, acenocoumarol)	High; unpredictable due to variable INR	Stop 4–5 days pre-operatively; check INR day before surgery	Only for very high thrombotic risk (mechanical mitral valve, recent VTE). Use LMWH.	Vitamin K 10 mg IV; 4-factor PCC for urgent reversal [76,77]
DOACs (Apixaban, Rivaroxaban, Edoxaban, Dabigatran)	Moderate to high; depends on renal function and drug accumulation	Stop 24–72 h pre-operatively based on renal function and bleeding risk of procedure	Not required	Idarucizumab (dabigatran) or Andexanet alfa (apixaban, rivaroxaban). 4-factor PCC as alternative [79]
P2Y12 Inhibitors (Clopidogrel, Ticagrelor, Prasugrel)	Moderate; irreversible platelet inhibition for 7–10 days	Stop 5–7 days pre-operatively for elective surgery	Not required. For recent stents (<1 month), discuss with cardiology.	Platelet transfusion. Note: ticagrelor may inhibit transfused platelets for up to 24 h [81]
NSAID (acetylsalicylic acid)	Low to moderate (monotherapy); higher when combined with P2Y12 inhibitor	Often continued in high cardiac risk patients; stop 5–7 days pre-operatively if bleeding risk is very high	Not required	Desmopressin (DDAVP) 0.3 μg/kg may be considered [82]

**Table 3:** Intraoperative PBM–Quick Reference for Common Scenarios (TXA—Tranexamic Acid, PPH—Postpartum Hemorrhage, Hb—Hemoglobin, ANH—Acute Normovolemic Hemodilution, TEG—Thromboelastography, ROTEM—Rotational Thromboelastometry, MAP—Mean Arterial Pressure, CAD—Coronary Artery Disease, CKD—Chronic Kidney Disease, FIBTEM—Fibrinogen Thromboelastometry, A5—Amplitude at 5 min, CT—Clotting Time, RT—Reaction Time, PCC—Prothrombin Complex Concentrate, FFP—Fresh Frozen Plasma, rFVIIa—Recombinant Activated Factor VII, DOAC—Direct Oral Anticoagulant, INR—International Normalized Ratio, TACO—Transfusion-Associated Circulatory Overload, DDAVP—Desmopressin (1-deamino-8-D-arginine vasopressin), VWF—Von Willebrand Factor, MTP—Massive Transfusion Protocol, PRBC—Packed Red Blood Cells, PROPPR—Pragmatic, Randomized Optimal Platelet and Plasma Ratios, LTOWB—Low-Titer Group O Whole Blood, TBI—Traumatic Brain Injury, SBP—Systolic Blood Pressure, DCR—Damage Control Resuscitation, REBOA—Resuscitative Endovascular Balloon Occlusion of the Aorta).

Clinical Scenario	Intraoperative Situation	First-Line Management	Second-Line	Special Considerations
Expected high blood loss (e.g., cardiac, major spine, liver resection)	Surgeon anticipates significant bleeding	• TXA 10–20 mg/kg before incision • Cell salvage set up and ready • Consider ANH if Hb adequate and center expertise	• Controlled hypotension (MAP 20–30% below baseline) if no CV/cerebrovascular disease	ANH is operationally demanding; not for every center. Cell salvage requires trained staff–know your team’s capabilities
Diffuse microvascular oozing	Surgical field looks “wet,” no major vessel bleeder	• Check temperature, calcium, pH (correct acidosis/hypothermia) • Topical hemostatic agent (fibrin sealant, gelatin-thrombin matrix)	• Consider TEG/ROTEM to rule out coagulopathy • Re-dose TXA if >3 h since last dose	Gelatin-thrombin matrices (Floseal) work well for irregular surfaces; fibrin sealants better for flat areas
Coagulopathy on TEG/ROTEM (prolonged clot time, reduced firmness)	Specific factor deficiency or fibrinogen problem	• Low fibrinogen (FIBTEM A5 < 10–12 mm) → fibrinogen concentrate 2–4 g (or cryoprecipitate) • Prolonged CT/RT → consider PCC if on warfarin/DOAC, otherwise FFP	• Persistent bleeding + prolonged CT after fibrinogen → 4-factor PCC (off-label but used) • rFVIIa–last resort (salvage only, thrombotic risk high)	Evidence for fibrinogen concentrate vs. cryo still mixed (FIBRES trial [87]). Use what you have, but give it early if indicated
Bleeding patient on antiplatelets (aspirin, clopidogrel, ticagrelor)	Platelet dysfunction ± recent stent	• For clopidogrel/prasugrel–platelet transfusion if last dose >6–12 h (but may be ineffective) • For ticagrelor–platelet transfusion often useless (active metabolite inhibits transfused platelets for ~24 h)	• Desmopressin (DDAVP) 0.3 mcg/kg–weak evidence, but some effect on bleeding time • TXA regardless	Involve cardiology early. If recent stent (<1 month), thrombotic risk is massive–balance is brutal
Massive transfusion activation (exsanguinating patient)	Trauma, ruptured AAA, obstetrics	• Activate MTP early (don’t wait for lab numbers) • 1:1:1 ratio (PRBC:FFP:platelets) as initial approach (PROPPR trial) • LTOWB if available	• Tranexamic acid 1 g over 10 min, then 1 g over 8 h (if within 3 h of injury) • Correct hypocalcemia (Ca++ > 1.2 mmol/L)–every unit of blood chelates calcium	Balanced resuscitation > pouring in crystalloid. Keep patient warm (every 1 °C drop increases transfusion needs ~20%). Permissive hypotension (SBP 80–90) until surgical control

**Table 4:** Clinical Pearls for Perioperative Blood Management (Hb, hemoglobin; IV, intravenous; DOAC, direct oral anticoagulant; LMWH, low-molecular-weight heparin; TEG, thromboelastography; ROTEM, rotational thromboelastometry; FIBTEM, fibrinogen thromboelastometry; MTP, massive transfusion protocol; PRBC, packed red blood cells; FFP, fresh frozen plasma; LTOWB, low-titer group O whole blood; TBI, traumatic brain injury).

Clinical Condition	Key Intervention
Preoperative screening	Screen every surgical patient for anemia (Hb < 13 g/dL men, <12 g/dL women) and measure ferritin, B12, and folate [15]
Iron deficiency	Use IV iron if surgery is <6 weeks away or if oral iron is poorly tolerated [19]
Tranexamic acid	Give 10–20 mg/kg before incision in any procedure with expected blood loss > 500 mL [28]
Anticoagulation	Bridging with LMWH is rarely needed (only for mechanical mitral valves or recent thrombosis). Stop DOACs 24–72 h preoperatively based on renal function [24]
Intraoperative	Use TEG/ROTEM for goal-directed coagulation therapy in high-risk cases (cardiac, trauma, liver). Target fibrinogen early (FIBTEM A5 < 10–12 mm) [35,156]
Transfusion	Use restrictive threshold of 7–8 g/dL for most stable patients. Exceptions: acute coronary syndrome (8–9 g/dL), brain injury (8–9 g/dL), ongoing bleeding (individualize) [9]
Massive Bleeding	Activate MTP early. Use 1:1:1 ratio (PRBC:FFP:platelets) or low-titer O whole blood if available. Keep patient warm and ionized calcium > 1.2 mmol/L [176,178]
Postoperatively	Continue anemia treatment (IV iron, B12/folate). Minimize iatrogenic blood loss [16]

## Data Availability

All data generated are available by the corresponding author upon request.

## Abbreviations

The following abbreviations are used in this manuscript:

AAA	Abdominal Aortic Aneurysm
ANH	Acute Normovolemic Hemodilution
aPTT	Activated Partial Thromboplastin Time
CABG	Coronary Artery Bypass Grafting
CAD	Coronary Artery Disease
CFCs	Coagulation Factor Concentrates
CKD	Chronic Kidney Disease
CPB	Cardiopulmonary Bypass
CT	Clotting Time (viscoelastic testing)
CV	Cardiovascular
CVA	Cerebrovascular Accident
DAPT	Dual Antiplatelet Therapy
DCR	Damage Control Resuscitation
DCS	Damage Control Surgery
DDAVP	Desmopressin (1-deamino-8-D-arginine vasopressin)
DOAC	Direct Oral Anticoagulant
EAST	Eastern Association for the Surgery of Trauma
ESA	Erythropoiesis-Stimulating Agent
ESAIC	European Society of Anaesthesiology and Intensive Care
ESC	European Society of Cardiology
FFP	Fresh Frozen Plasma
FIBTEM	Fibrinogen Thromboelastometry (ROTEM assay)
GDFT	Goal-Directed Fluid Therapy
GIHP	French Working Group on Perioperative Haemostasis
Hb	Hemoglobin
IBD	Inflammatory Bowel Disease
ICS	Intraoperative Cell Salvage
ICU	Intensive Care Unit
IDA	Iron Deficiency Anemia
INR	International Normalized Ratio
IV	Intravenous
LMWH	Low-Molecular-Weight Heparin
LOS	Length of Stay
LTOWB	Low-Titer Group O Whole Blood
MAP	Mean Arterial Pressure
MIS	Minimally Invasive Surgery
MTP	Massive Transfusion Protocol
PAD	Preoperative Autologous Donation
PBM	Patient Blood Management
PCC	Prothrombin Complex Concentrate
POCT	Point-of-Care Testing
PPH	Postpartum Hemorrhage
PRBC	Packed Red Blood Cells
PROPPR	Pragmatic, Randomized Optimal Platelet and Plasma Ratios trial
REBOA	Resuscitative Endovascular Balloon Occlusion of the Aorta
rFVIIa	Recombinant Activated Factor VII
ROTEM	Rotational Thromboelastometry
RT	Reaction Time (viscoelastic testing)
SBP	Systolic Blood Pressure
TACO	Transfusion-Associated Circulatory Overload
TBI	Traumatic Brain Injury
TEG	Thromboelastography
TRALI	Transfusion-Related Acute Lung Injury
TRICC	Transfusion Requirements in Critical Care trial
TRICS	Transfusion Requirements in Cardiac Surgery trial
TRIM	Transfusion-Related Immunomodulation
TXA	Tranexamic Acid
VWD	Von Willebrand Disease
VWF	Von Willebrand Factor
